# Conserved surface-exposed linear epitopes of *Echinococcus multilocularis* thioredoxin peroxidase enable non-invasive diagnosis of alveolar echinococcosis in intermediate host

**DOI:** 10.1371/journal.pntd.0014755

**Published:** 2026-09-22

**Authors:** Lijuan Zheng, Wenjing Zhi, Mengjie Chu, Xinyi Zhou, Yong Fu, Chen Xin, Baoping Li, Lingzhi Pengmao, Zhendong Xin, Yihui Wu, Xuejun Ji, Anni Zhang, Changjie Qian, Zhen Wang, Chenye Jin, Yun Han, Zhijun Zhang, Fuyun Chen, Gongguan Liu

**Affiliations:** 1 Key Laboratory of Animal Disease Diagnostics and Immunology, Ministry of Agriculture, MOE International Joint Collaborative Research Laboratory for Animal Health & Food Safety, College of Veterinary Medicine, Nanjing Agricultural University, Nanjing, China; 2 Academy of Animal Science and Veterinary, Qinghai Provincial Key Laboratory of Pathogen Diagnosis for Animal Diseases and Green Technical Research for Prevention and Control, Qinghai University, Xining, China; 3 The Haixi Prefecture Center for Animal Disease Control and Prevention, Delingha, China; 4 The Wulan County Center for Disease Control and Prevention, Wulan, China; Wellcome Sanger Institute, UNITED KINGDOM OF GREAT BRITAIN AND NORTHERN IRELAND

## Abstract

Alveolar echinococcosis (AE) is a severe zoonotic parasitic disease caused by the larval stage of *Echinococcus multilocularis*, for which early and accurate diagnosis remains challenging. In this study, we targeted *E. multilocularis* thioredoxin peroxidase (EmTPx), a highly abundant excretory/secretory protein conserved across the genus *Echinococcus*, for diagnostic development. Monoclonal antibodies (mAbs) against EmTPx were generated, and two linear B‑cell epitopes, ^174^HPGSK^178^ and ^189^SFMSS^193^, were identified. Both epitopes are strictly conserved within *Echinococcus* but markedly divergent from mammalian host sequences. Structural modeling revealed that the epitopes are surface‑exposed, with ^174^HPGSK^178^ located in a flexible loop and ^189^SFMSS^193^ adjacent to an α‑helix, conformations favorable for antibody recognition. Two ELISA methods were established using the epitope‑specific mAbs. The blocking ELISA showed moderate diagnostic performance. More importantly, the sandwich ELISA enabled direct detection of EmTPx antigen in fecal samples of intermediate hosts, revealing a detection rate of 85% in mouse feces at an early infection stage. With a linear range of 0–250 ng/mL, a cutoff of 0.1724, and intra‑ and inter‑assay precision below 10%, this sandwich ELISA demonstrated robust analytical performance. In conclusion, this proof-of-concept study identifies two evolutionarily conserved and structurally exposed EmTPx epitopes and demonstrates their potential utility for the development of monoclonal antibody-based approaches for non-invasive detection of echinococcosis.

## Author summary

Alveolar echinococcosis (AE) is a life-threatening parasitic disease caused by the larval stage of *Echinococcus multilocularis*. Early diagnosis remains challenging, as current blood-based tests detect host antibodies but often fail in immunocompromised individuals and cannot reliably distinguish active from past infection. In this study, we developed a non-invasive diagnostic approach that directly detects a parasite excretory/secretory protein called thioredoxin peroxidase (EmTPx) in fecal samples. We generated monoclonal antibodies targeting two specific regions of EmTPx that are highly conserved among *Echinococcus* species yet markedly different from mammalian TPx homologs, ensuring specific recognition of the parasite. Using these antibodies, we constructed a sandwich ELISA and successfully detected EmTPx in the feces of experimentally infected mice at an early stage of infection. This finding supports a mechanistic model in which parasite proteins released from hepatic lesions reach the intestinal lumen through hepatobiliary transport, thereby enabling non-invasive fecal detection. Beyond providing a theoretical foundation for discovering additional coproantigen targets, this approach offers a promising tool for early diagnosis and large-scale screening of AE, particularly in resource-limited endemic settings where current diagnostic methods are unavailable or insufficient.

## Introduction

Alveolar echinococcosis (AE) is a life‑threatening zoonotic parasitic disease caused by the larval stage of *E. multilocularis*. The metacestode resides predominantly in the liver, where it undergoes infiltrative, tumor-like proliferation, progressively invading adjacent tissues with the potential to colonize distant organs [1–3]. If left untreated, AE can lead to severe hepatic dysfunction and even death [1]. Early and accurate diagnosis is therefore critical for effective clinical management. However, the asymptomatic onset of early‑stage infection often delays diagnosis [1], such that most cases were identified only after substantial lesion development, significantly compromising treatment outcomes and prognosis [4].

Currently, the diagnosis of AE primarily relies on imaging examinations combined with serological tests. Ultrasound is widely used for screening due to its non-invasiveness and accessibility [2,5]. Computed Tomography (CT) and Magnetic Resonance Imaging (MRI) offer higher‑resolution structural information that supports detailed evaluation and differential diagnosis. However, these imaging modalities generally detect the disease only after typical lesions have developed, limiting their utility for early diagnosis and their accessibility in resource-limited settings [5]. Serological assays, particularly ELISA, are widely used for AE diagnosis because of their simplicity and suitability for large-scale screening [6,7]. However, variable antibody responses, reduced sensitivity in immunocompromised individuals, and the persistence of antibodies after treatment limit their ability to accurately reflect active infection [8].

Antigen detection represents a particularly attractive approach because parasite-derived molecules are more closely associated with ongoing biological activity than host antibody responses, which may more accurately reflect active infection [9]. In particular, monoclonal antibody (mAb)-based immunoassays offer high analytical specificity and reproducibility. The dual-epitope sandwich ELISA, by simultaneously recognizing two non-overlapping epitopes, provides superior sensitivity and specificity for directly detecting parasite-derived molecules in biological samples such as serum and feces [10–12]. Therefore, the identification of suitable parasite-derived antigens that are abundantly expressed, actively secreted, and readily accessible in host biological samples has become a critical prerequisite for the development of next-generation diagnostic tools for AE.

Thioredoxin peroxidase (TPx) is a member of the peroxiredoxin family [13,14]. As a key antioxidant enzyme, TPx catalyzes the reduction of peroxides to maintain redox homeostasis [15], enabling parasites to withstand host-derived reactive oxygen species. Previous studies have demonstrated that TPx proteins are highly expressed and conserved among cestodes and can interact with the host immune system, potentially modulating macrophage polarization and other immune responses [16,17]. Recent studies have shown that, in several parasitic helminths, TPx proteins are not restricted to intracellular antioxidant functions but can also be released into the host environment as excretory/secretory products (E/SPs), including in *Schistosoma*, *Fasciola*, and *Clonorchis* species [18]. These characteristics suggest that EmTPx may represent a promising antigen for parasite detection; however, its diagnostic potential in AE has not yet been systematically investigated. This knowledge gap motivated the development of EmTPx-targeted diagnostic tools in the present study.

In this study, we hypothesize that EmTPx contains species-specific epitopes amenable to non-invasive immunodiagnostic development. To this end, we generated a panel of mAbs against EmTPx and mapped two distinct linear epitopes suitable for immunoassay development. Using these antibodies, we established both a blocking ELISA and a dual-epitope sandwich ELISA, and evaluated their diagnostic performance in naturally and experimentally infected hosts. Our findings provide a basis for the development of EmTPx-based coproantigen detection approaches and support their potential utility for non-invasive surveillance and early-stage detection of *E. multilocularis* infections.

## Materials and methods

### Ethics statement

All animal experimental procedures were conducted in strict accordance with the Regulations for the Administration of Laboratory Animals issued by the Ministry of Science and Technology of the People's Republic of China, as well as the guidelines of the Institutional Animal Care and Use Committee (IACUC) of Nanjing Agricultural University. The study protocol was reviewed and approved by the Animal Ethics Committee of Nanjing Agricultural University under approval No. NJAU.No20260303033.

### Cells, plasmids and animals

Murine myeloma SP2/0 cells were maintained in our laboratory and cultured in RPMI-1640 medium supplemented with 15% fetal bovine serum (FBS) at 37°C in a humidified incubator with 5% CO_2_. *Escherichia coli* strains DH5α and BL21 (DE3) were purchased from Tsingke Biotechnology Co., Ltd. The expression vectors pET-28a and pET-32a were preserved in our laboratory. ICR and BALB/c mice (6–8 weeks old, female) were obtained from the Experimental Animal Center of Yangzhou University. All animals were maintained under specific pathogen-free (SPF) conditions.

### Gene cloning, prokaryotic expression and purification

The nucleotide and amino acid sequences of EmTPx (GenBank accession No. AB071135.1; UniProt accession No. Q86M52) and homologous peroxiredoxin proteins from other parasite species were retrieved from public databases, including GenBank, UniProt, and WormBase Parasite. Multiple sequence alignment and sequence analyses were performed using the obtained protein sequences. Primers were designed based on the *E. multilocularis* TPx genomic sequence. All primers were synthesized by Sangon Biotech Corporation (Shanghai, China), and their sequences are listed in Table 1. The full-length EmTPx gene was cloned into pET-28a for recombinant protein production, while truncated EmTPx fragments for epitope mapping were cloned into pET-32a to facilitate soluble expression of short peptide fragments. Following confirmation by colony PCR and Sanger sequencing, the positive recombinant plasmid pET‑28a‑TPx was transformed into *E. coli* BL21 (DE3) for protein expression. When the bacterial culture reached an OD600 of 0.6–0.8, protein expression was induced with 0.5 mM isopropyl β-D-1-thiogalactopyranoside (IPTG) at 37°C for 6 hours. The supernatant and inclusion body fractions of the induced bacteria were collected and analyzed by colony PCR and Western blotting. The recombinant protein was subsequently purified using high‑affinity Ni‑NTA resin.

**Table 1:** Primers sequences for truncated EmTPx proteins.

Amplified region	Primer	Sequence (5’-3’)
1-193aa	TPx-F	GGTCGCGGATCCGAATTCATGGTTGCTCTTGTTGGG
	TPx-R	GTGGTGGTGGTGCTCGAGCGAGCTCATGAACGACTT
1-70 aa	TPx-F1-F	ATCGGATCCGAATTCATGGTTGCTCTTGTT
	TPx-F1-R	GTGGTGGTGCTCGAGAAGGAGCTGGCACCC
61-132 aa	TPx-F2-F	ATCGGATCCGAATTCGAGTTTCGTCAGCGT
	TPx-F2-R	GTGGTGGTGCTCGAGCTTGTCGTCGATGAT
123-193aa	TPx-F3-F	ATCGGATCCGAATTCCTCCGCGGCCTTTTTATC
	TPx-F3-R	GTGGTGGTGCTCGAGCGAGCTCATGAACGA
123-152 aa	TPx-F4-F	ATCGGATCCGAATTCCTCCGCGGCCTTTTTATC
	TPx-F4-R	GTGGTGGTGCTCGAGAGCCTCATCCACAGAGCG
143-173 aa	TPx-F5-F	ATCGGATCCGAATTCTTGCCCGTCGGCCGC
	TPx-F5-R	GTGGTGGTGCTCGAGCCAATTTGCTGGGCAAAC
164-193 aa	TPx-F6-F	ATCGGATCCGAATTCAAGCATGGTGAGGTT
	TPx-F6-R	GTGGTGGTGCTCGAGCGAGCTCATGAACGACTT
174-188 aa	TPx-F7-F	ATCGGATCCGAATTCCACCCTGGATCAAAAACC
	TPx-F7-R	GTGGTGGTGCTCGAGCTTCAAATCACCCGC
179-193 aa	TPx-F8-F	ATCGGATCCGAATTCACCTTCAAACCGAGTGCG
	TPx-F8-R	GTGGTGGTGCTCGAGCGAGCTCATGAACGACTT
143-178aa	TPx-F9-F	ATCGGATCCGAATTCTTGCCCGTCGGCCGC
	TPx-F9-R	GTGGTGGTGCTCGAGTTTTGATCCAGGGTG

aa, amino acid residues.

^a^The table presents the primers used for amplification of the full-length EmTPx gene and its truncated fragments, including the corresponding amplified amino acid regions.

Primers sequences are presented in the 5’–3’ direction.

### Data-independent acquisition mass spectrometry (DIA-MS)

Cyst fluid samples from *E. multilocularis* infected mice were digested with trypsin and analyzed by nano DIA-MS using an Orbitrap Astral high-resolution mass spectrometer in Data Independent Acquisition (DIA) mode. Peptides were separated on a reverse-phase analytical column using a linear acetonitrile gradient and detected within a predefined mass range. Internal retention time (iRT) peptides were added for cross-batch retention time calibration. Raw DIA data were processed using a corresponding DIA analysis platform against the *E. multilocularis* reference database for peptide identification and protein quantification.

### Analysis of EmTPx protein

Secondary structure prediction of EmTPx was performed using the DTU Health Tech server (NetSurfP 3.0 - DTU Health Tech - Bioinformatic Services) to evaluate the distribution of α-helices, β-strands, and random coils. Linear B-cell epitopes were predicted using BepiPred-2.0 [19] (BepiPred 2.0 - DTU Health Tech - Bioinformatic Services) with a default threshold of 0.15. Sequence conservation analysis was performed by multiple sequence alignment of EmTPx with homologous TPx sequences from representative parasitic species, including *Echinococcus granulosus*, *Taenia solium*, *Clonorchis sinensis*, and *Fasciola hepatica*, using the MUSCLE algorithm implemented in MEGA 11 with default parameters [20]. The alignments were visualized and manually inspected using Jalview [21]. To investigate the evolutionary relationship between thioredoxin peroxidase (EmTPx) from *E. multilocularis* and peroxidase proteins from other cestode species, a phylogenetic analysis was performed. Amino acid sequences of peroxidase proteins from cestodes were retrieved from the NCBI database, including *Echinococcus granulosus*, *Taenia solium*, *Taenia crassiceps*, *Taenia multiceps*, *Hydatigera taeniaeformis*, *Hymenolepis microstoma*, *Hymenolepis diminuta*, *Taenia ovis*, and *Diphyllobothrium latum*. A phylogenetic tree was constructed using the Maximum Likelihood method implemented in MEGA version 11, with 1,000 bootstrap replicates to assess branch support. The resulting tree was visualized and annotated.

### Immunization of animals

Three female BALB/c mice (8 weeks old) were immunized with recombinant EmTPx protein following the immunization protocol described below. The recombinant protein was emulsified with an equal volume of Freund’s complete adjuvant and administered subcutaneously at multiple dorsal sites on the back at a dose of 80 μg per mouse for the primary immunization. Two weeks later, the same dose emulsified with Freund’s incomplete adjuvant was administered for the second immunization, and a third immunization was performed two weeks after the second dose.

Ten days after the third immunization, antibody titers in mouse sera were measured by indirect ELISA, and the mouse exhibiting the highest antibody level was selected for booster immunization. This mouse received an intraperitoneal injection of 50 μg recombinant EmTPx to enhance the proportion of antibody‑producing splenocytes. Three days later, spleens were collected for cell fusion.

### Preparation of monoclonal antibodies

Three days after the booster immunization, the mouse was euthanized and splenocytes were harvested. After counting, splenocytes were fused with SP2/0 myeloma cells at a ratio of 1:7 using PEG4000. The fused cells were seeded into 96‑well plates containing RPMI‑1640 medium supplemented with 15% FBS and 2% HAT. After 10 days, the culture supernatant was replaced with RPMI‑1640 containing 15% FBS and 2% HT. Forty‑eight hours later, hybridoma supernatants were screened by indirect ELISA, and positive wells were selected for subsequent rounds of subcloning. After at least three rounds of limiting dilution, monoclonal hybridoma lines with high titers and stable growth were expanded.

Following expansion for at least 20 passages, hybridoma cells that consistently secreted monoclonal antibodies were injected intraperitoneally into 8‑week‑old female BALB/c mice pre‑primed with liquid paraffin to induce ascites production. Antibody titers in ascites were determined by indirect ELISA. The specificity of the monoclonal antibodies was evaluated by Western blotting, immunofluorescence assay (IFA), and immunohistochemistry (IHC). The heavy‑ and light‑chain isotypes of the monoclonal antibodies were identified using a commercial isotyping kit (Proteintech, Wuhan, China; Cat: PK20002).

### Mapping of epitope targeted by mAb

To characterize the minimal linear epitopes recognized by the EmTPx monoclonal antibodies, nine primer pairs were designed to amplify truncated TPx fragments. These fragments were generated by PCR, cloned into the pET‑32a vector, and expressed as recombinant truncated proteins in *E. coli* BL21 (DE3). Epitope mapping was performed by Western blotting using the monoclonal antibodies. Multiple sequence alignment was conducted using MEGA 11 to evaluate the conservation of the core epitopes across species. The predicted three‑dimensional structure of EmTPx (AlphaFold Server) [22] was visualized using PyMOL [23], and the positions of the core epitopes were located and highlighted within the structural model.

### SDS-PAGE and Western blot

Protein samples or protoscolex lysates were separated by electrophoresis on 12% SDS–polyacrylamide gels. Gels were either stained with Coomassie Brilliant Blue or transferred onto nitrocellulose membranes. The membranes were blocked with TBST containing 5% skim milk and subsequently incubated overnight at 4°C with the appropriate primary antibodies. After three washes with TBST, the membranes were incubated with horseradish peroxidase (HRP)‑conjugated secondary antibodies at room temperature for 1 hour, followed by three additional washes. Protein bands were visualized using an ECL detection reagent, and images were captured using a Tanon automated chemiluminescence imaging system according to the manufacturer's instructions.

### Ascites purification

Ascites-derived monoclonal antibodies were purified by Protein G affinity chromatography (GenScript,Cat: L00664). Ascites samples were incubated with Protein G resin to capture immunoglobulins, followed by column loading and washing to remove unbound proteins. Bound antibodies were eluted under acidic conditions and immediately neutralized. The purity of the purified antibodies was assessed by Coomassie Brilliant Blue staining. Purified antibodies were subsequently subjected to buffer exchange and concentration prior to downstream applications. Detailed purification procedures and buffer formulations are described in the Supplementary Information file.

### HRP labeling of monoclonal antibodies

The EmTPx monoclonal antibodies were conjugated to horseradish peroxidase (HRP) using a rapid HRP‑labeling kit (Bioablab,Cat: 6210). A total of 500 μL antibody solution (0.5 mg) was mixed thoroughly with 100 μL of pre‑activated HRP and 108 μL of labeling initiator (added at 1.8 μL per 10 μL of the antibody–HRP mixture). The reaction mixture was incubated at 4°C for 24 hours. Subsequently, 100 μL of the stop solution was added, and the mixture was incubated at room temperature for an additional 2 hours. An equal volume of storage buffer was then added to complete the conjugation process. The HRP‑labeled antibodies were stored at −20°C until use.

### Indirect immunofluorescence assay (IFA)

The localization of EmTPx in the larval stage of *E. multilocularis* was determined by immunofluorescence. Fresh PSCs were collected from experimentally infected gerbils and washed three times with phosphate‑buffered saline (PBS) to remove host‑derived contaminants. The PSCs were fixed in 4% paraformaldehyde at room temperature for 15 minutes, followed by three additional washes with PBS. Fixed samples were permeabilized with 0.3% Triton X‑100 in PBS for 15 minutes and subsequently blocked with PBS containing 5% bovine serum albumin (BSA) for 2 hours at room temperature to minimize nonspecific binding.

Blocked PSCs were incubated overnight at 4°C with mouse monoclonal anti‑EmTPx antibodies diluted 1:100 in PBS containing 1% BSA. After three washes with PBS, the samples were incubated with fluorescein isothiocyanate (FITC)‑conjugated goat anti‑mouse IgG (1:2000 dilution) for 2 hours at room temperature in the dark. Following additional PBS washes, nuclei were counterstained with Nuclear Green for 10 minutes. Finally, the PSCs were mounted on glass slides using an anti‑fade reagent and examined under a Nikon confocal microscope, and images were captured using Nikon imaging software.

### Screening of antibody pairs for sandwich ELISA

Two distinct antigenic epitopes of EmTPx were identified, and two monoclonal antibodies were obtained for each epitope based on isotype analysis. To ensure effective antibody pairing for the development of the sandwich ELISA, one representative monoclonal antibody from each isotype group corresponding to the two epitopes was selected, resulting in a total of four monoclonal antibodies (1G2A3, 5H8A8, 3E2D11, and 4E4E3) for subsequent pairing experiments.

Recombinant EmTPx protein was used as the positive antigen control, while PBS was included as the negative control. The antibody pairing efficiency was assessed by comparing the OD450 values between positive and negative wells, and the optimal capture–detection antibody combination was selected based on the highest positive-to-negative (P/N) ratio. Detailed antibody concentrations, incubation conditions, and optimization procedures are provided in the Supplementary Information file.

### Establishment and optimization of the blocking ELISA method

A blocking ELISA was established using recombinant EmTPx as the coating antigen and EmTPx-specific monoclonal antibodies as detection antibodies. Initially, nine monoclonal antibodies generated in this study were evaluated for their ability to inhibit antibody binding using positive and negative sera. The inhibition rate (PI) was calculated according to the following formula: PI (%) = [(OD450 of negative control − OD450 of test sample) / OD450 of negative control] × 100%. The monoclonal antibody showing the highest inhibition rate was selected for subsequent assay optimization. Key assay parameters, including antigen coating concentration, monoclonal antibody dilution, blocking conditions, sample incubation time, antibody incubation time, and substrate development time, were systematically optimized. The optimal conditions were determined based on the highest inhibition rate. Detailed optimization procedures are provided in the Supplementary Information file.

Using the optimized assay conditions, 22 positive sheep sera and 65 negative sera that were collected from animals showing typical gross lesions at a slaughterhouse were tested, and inhibition rates were calculated. Receiver operating characteristic (ROC) curves were generated using GraphPad Prism 10.4.0. The optimal cutoff value, sensitivity, and specificity were determined using the maximum Youden index, calculated as sensitivity + specificity − 1. The inhibition rate corresponding to the maximum Youden index was defined as the optimal cutoff. Sample sizes were determined based on the objectives of assay development and preliminary validation, considering sample availability, ethical requirements, and the need to assess diagnostic performance.

### Optimization of sandwich ELISA procedure

Using purified 3E2D11 as the capture antibody and HRP‑conjugated 5H8A8 (HRP‑5H8A8) as the detection antibody, the conditions for the sandwich ELISA were systematically optimized. A checkerboard titration was first performed to determine the optimal coating concentration of 3E2D11 (0.5, 1.0, 2.0, 4.0, 8.0, 16.0 μg/mL) and the working dilution of HRP‑5H8A8 (1:1000, 1:2000, 1:4000, 1:8000, 1:16000, 1:32000). Subsequent optimization included sequential evaluation of blocking conditions (1%, 2.5%, or 5% BSA or skim milk, with incubation times ranging from 0.5 to 2 hours at 0.5‑hour intervals), serum dilution ratios (1:1, 1:5, 1:10, 1:20), recombinant antigen incubation time (0.5–2 hours), HRP‑5H8A8 incubation time (0.5–2 hours), and TMB substrate reaction time (5, 10, 15, or 20 minutes). For each parameter, the optimal condition was determined based on the OD450 ratio between positive and negative samples.

### Performance verification of sandwich ELISA

Fecal samples were collected from mice experimentally infected with *E. multilocularis* protoscoleces at 6 weeks post-infection (positive group) and from uninfected mice (negative group). A total of 20 fecal samples from infected mice and 32 fecal samples from uninfected mice were analyzed.

Under the optimized assay conditions, 32 negative fecal samples were tested, and the cutoff value was determined as the sum of the mean OD and three times the standard deviation. Six fecal samples were analyzed in triplicate on a single plate to assess intra-assay variation, while inter‑assay variation was evaluated across three independent plates. The CV was calculated from the mean and standard deviation to verify assay precision.

For quantitative analysis, recombinant EmTPx was serially two‑fold diluted in PBS from 4000 ng/mL to 0.122 ng/mL. A standard curve was generated by plotting EmTPx concentration against OD450 values to assess linear detection capability. To assess the performance of the sandwich ELISA, a comparative analysis was performed using fecal samples from experimentally infected mice at 6 weeks post-infection with *E. multilocularis*. The limit of detection (LOD) and limit of quantification (LOQ) were determined as the mean OD450 of 32 negative fecal samples plus three and ten standard deviations (SD), respectively. The corresponding antigen concentrations were calculated using the standard curve.

## Results

### Proteomic identification and bioinformatic characterization of EmTPx as a candidate diagnostic antigen

Mass spectrometry analysis of *E. multilocularis*–derived proteins identified EmTPx as markedly enriched (Fig 1A), indicating its stable presence in infection-associated microenvironments. Sequence alignment revealed high conservation with its *E. granulosus* ortholog (Fig 1B) but limited homology to host TPx proteins (Fig 1C), suggesting parasite-specific antigenic characteristics. Further phylogenetic reconstruction (Fig 1D) placed EmTPx in a robustly supported monophyletic clade with *E. granulosus* TPx, confirming tight evolutionary conservation within *Echinococcus*, while TPx proteins from *Taenia* and *Hymenolepis* formed distinct lineages and *Sparganum proliferum* occupied an early-diverging position.

**Fig 1:**
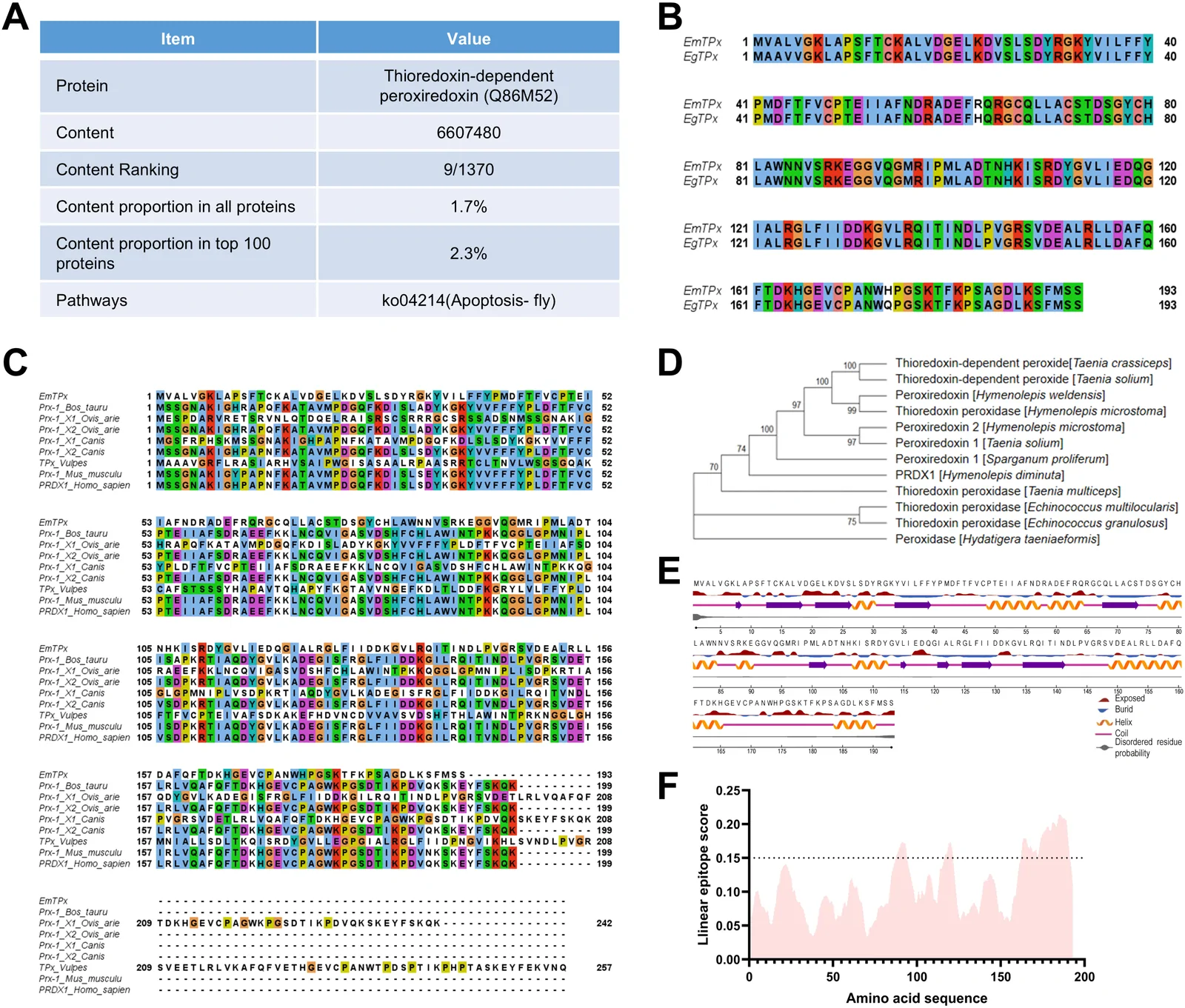
Antigen screening and characterization of EmTPx. **(A)** Identification of EmTPx based on proteomic analysis of *E. multilocularis* cyst fluid, supporting its selection as a candidate diagnostic antigen. **(B)** Sequence alignment revealing a high degree of sequence similarity between EmTPx and EgTPx. **(C)** Homology analysis between EmTPx and TPx homologs from common mammalian hosts of *E. multilocularis*, showing relatively low sequence similarity between parasite EmTPx and host TPx homologs. **(D)** Phylogenetic analysis of TPx homologs from different cestode species, illustrating the evolutionary relationship of EmTPx with other cestode TPx proteins. **(E)** Prediction of the structural features of EmTPx, including exposed, buried, helix, and coil regions. **(F)** Linear B‑cell epitope prediction of EmTPx using BepiPred‑2.0.

Secondary structure prediction revealed a predominance of α-helices and β-strands interspersed with flexible coils (Fig 1E), potentially facilitating substrate or host protein interactions. BepiPred 2.0 analysis identified several high-scoring linear B-cell epitopes (>0.15) within regions 88–94, 117–121, and 163–193 (Fig 1F), representing accessible antigenic determinants that provide a rational basis for peptide antigen design and monoclonal antibody development. Collectively, these findings confirm the high abundance, immunogenicity, and conserved accessibility of EmTPx, supporting its suitability as a diagnostic antigen target.

### Expression and purification of EmTPx protein

The EmTPx gene consists of 582 bp and encodes a 193-amino acid protein (UniProt accession No. Q86M52). The recombinant EmTPx protein was successfully expressed (Fig 2A). As expected, the recombinant protein exhibited an approximate molecular weight of 25 kDa (Fig 2B). SDS-PAGE analysis of the purified protein revealed a prominent band around 25 kDa, with markedly enhanced purity at imidazole concentrations of 300, 400, and 500 mM (Fig 2C). Importantly, sera from infected mice and sheep recognized the recombinant EmTPx protein, demonstrating its antigenicity (Fig 2D).

**Fig 2:**
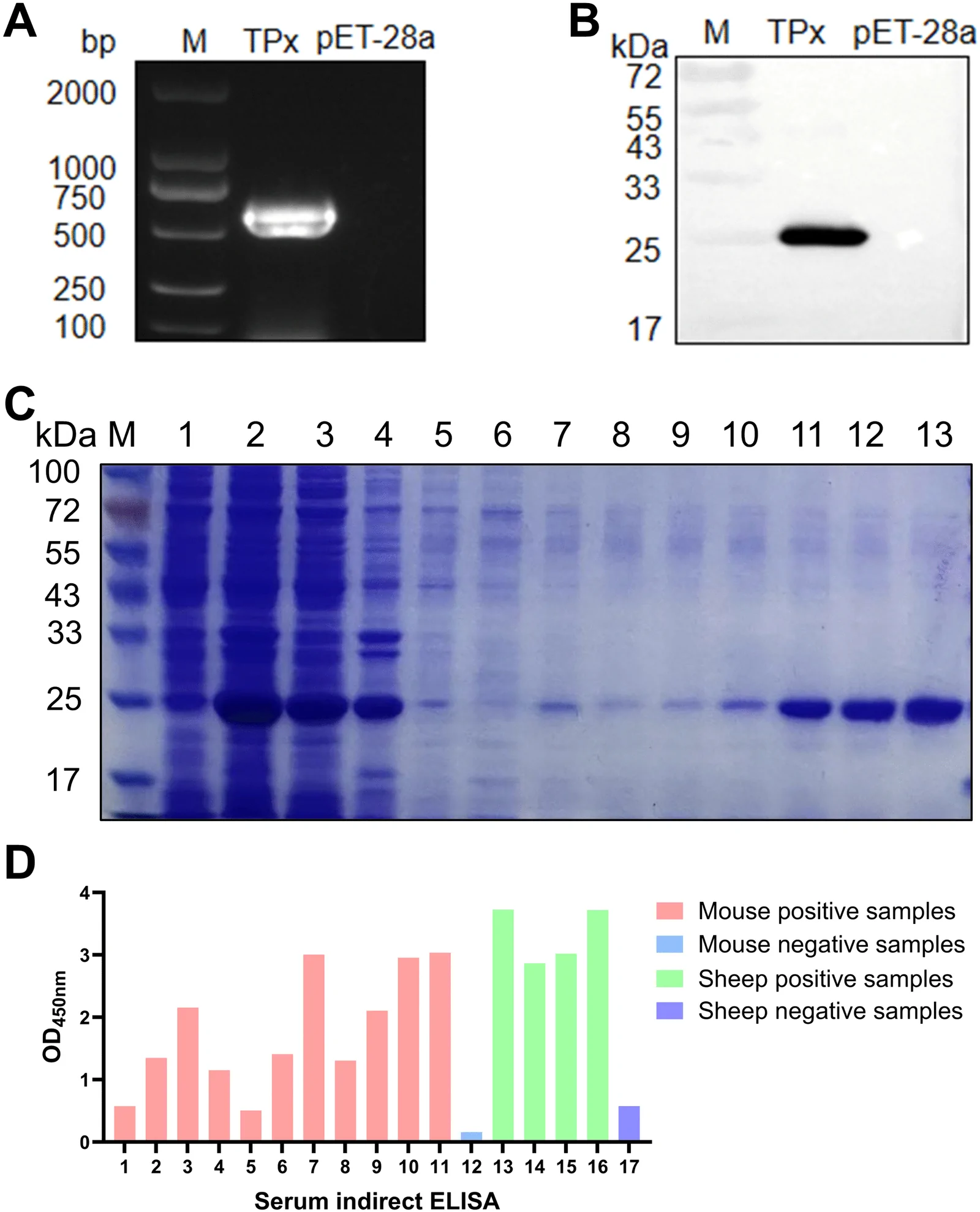
Expression and purification of His‑tagged recombinant EmTPx protein. **(A)** PCR amplification of the EmTPx gene. Lane M, DNA marker; lane 1, PCR product of EmTPx; lane 2, negative control. **(B)** Expression of recombinant EmTPx in *E. coli* under 0.5 mM IPTG induction, analyzed by Western blot using anti‑His monoclonal antibody. Lane M, protein molecular weight marker; lane 1, pET‑28a‑TPx recombinant protein; lane 2, pET‑28a empty vector. **(C)** Protein purification results. M: Molecular weight marker; 1: Flow-through; 2–4: Induced whole-cell, supernatant, precipitate; 5–13: 20, 40, 60, 80, 100, 200, 300, 400, 500 mM imidazole elution buffer. **(D)** Indirect ELISA using positive and negative sera from mice and sheep.

### Preparation of monoclonal antibody against EmTPx

The workflow for monoclonal antibody (mAb) generation is illustrated in Fig 3A. Mice immunized with purified recombinant EmTPx developed markedly elevated serum antibody titers compared with non-immunized controls (Fig 3B), confirming that the recombinant protein elicited a robust humoral immune response.

**Fig 3:**
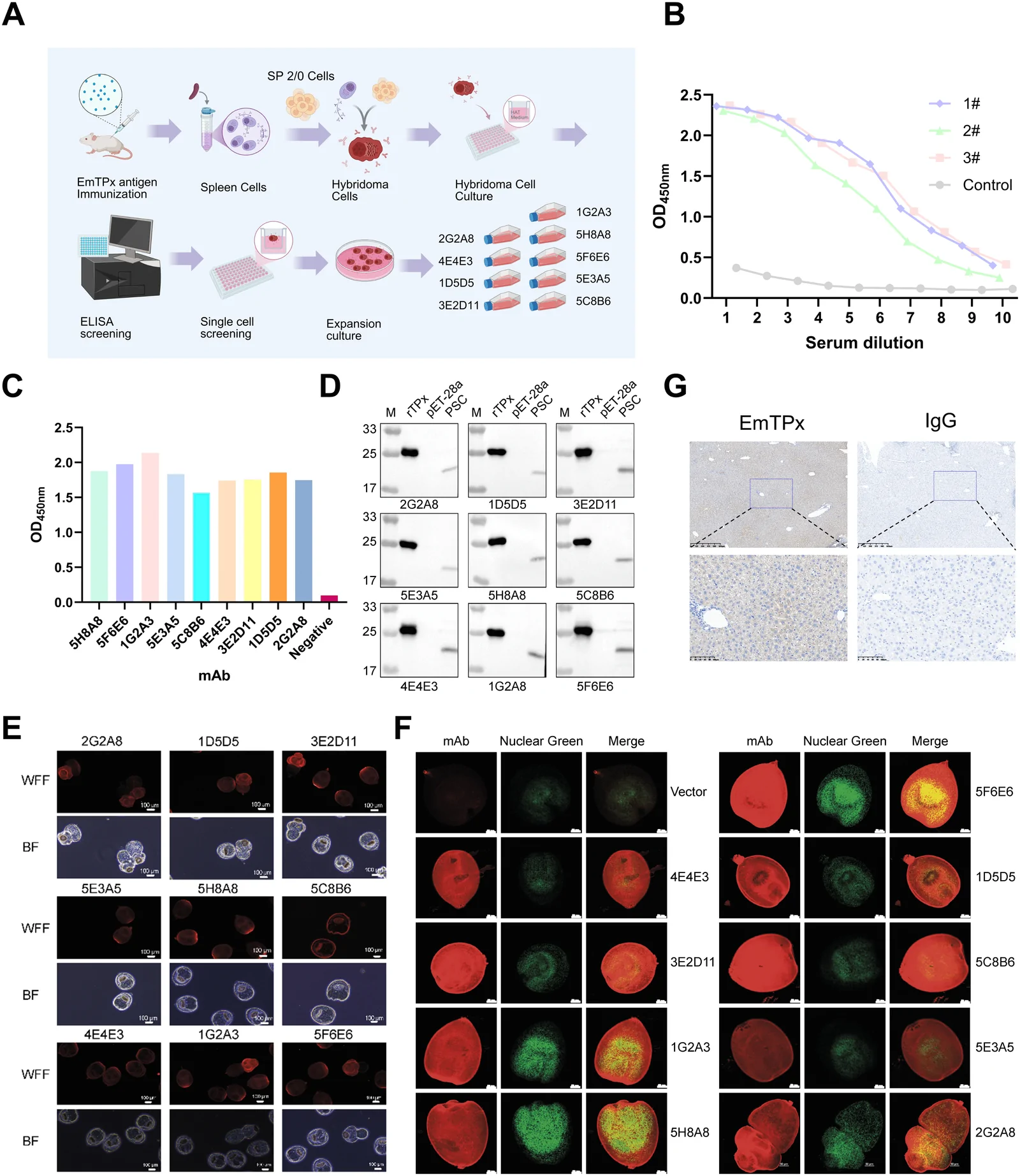
Production and characterization of monoclonal antibodies against EmTPx. **(A)** Preparation process of monoclonal antibodies (mAbs). Created in BioRender. Liu, G. (2026) https://BioRender.com/99erjyb **(B)** Indirect ELISA of mouse sera collected after the final booster immunization. Serial two‑fold dilutions of sera were tested against purified recombinant EmTPx protein. Sera from unimmunized mice were tested in parallel as negative controls. Antibody titers are expressed as absorbance values at 450 nm. **(C)** Indirect ELISA screening of culture supernatants from nine hybridoma clones using purified recombinant EmTPx protein as the coating antigen. Supernatant from SP2/0 cells served as the negative control. Antibody titers are expressed as absorbance values at 450 nm. **(D)** Western blot analysis of the nine mAbs. Lane M, protein molecular weight marker; lane 1, pET‑28a‑EmTPx recombinant protein; lane 2, pET‑28a empty vector; lane 3, protoscolex lysate. **(E)** Immunofluorescent staining of PSCs using EmTPx mAbs. BF, Bright field imaging; WFF, Wide field fluorescence imaging. **(F)** Immunoconfocal analysis of PSCs incubated with EmTPx mAbs. **(G)** Immunohistochemical staining of liver cyst samples from *E. multilocularis*-infected mice using EmTPx mAb and IgG antibody.

Nine hybridoma cell lines stably secreting EmTPx-specific mAbs were obtained, namely 5H8A8, 5F6E6, 5C8B6, 1G2A3, 5E3A5, 4E4E3, 1D5D5, 2G2A8, and 3E2D11 (Fig 3C). These hybridomas maintained stable antibody secretion after 20 passages, with titers ranging from 1:12,800–1:51,200, and all mAbs contained kappa light chains, among which 1G2A3 and 3E2D11 were classified as IgG2b while the remaining seven were IgG1 (Table 2).

**Table 2:** Titers and subtype mAbs.

mAbs	Titers in cell culture supernatant				Titers in ascites	HeavyChain	LightChain
	F5	F10	F15	F20			
5H8A8	1:25600	1:25600	1:25600	1:25600	1:1024000	IgG1	Kappa
5F6E6	1:51200	1:51200	1:51200	1:51200	1:1024000	IgG1	Kappa
2G2A8	1:12800	1:12800	1:12800	1:12800	1:1024000	IgG1	Kappa
1D5D5	1:51200	1:51200	1:102400	1:102400	1:256000	IgG1	Kappa
4E4E3	1:25600	1:25600	1:25600	1:25600	1:1024000	IgG1	Kappa
5C8B6	1:51200	1:51200	1:51200	1:102400	1:16000	IgG1	Kappa
5E3A5	1:12800	1:12800	1:12800	1:12800	1:1024000	IgG1	Kappa
3E2D11	1:25600	1:25600	1:25600	1:25600	1:8000	IgG2b	Kappa
1G2A3	1:51200	1:51200	1:51200	1:51200	1:64000	IgG2b	Kappa

mAb, monoclonal antibody; IgG, immunoglobulin G; Kappa, kappa light chain.

^a^Antibody titers of hybridoma culture supernatants and ascites were determined by indirect ELISA. F5, F10, F15, and F20 indicate different passages of hybridoma cultures. Heavy- and light-chain types of the monoclonal antibodies are also indicated.

The EmTPx mAbs specifically recognized both recombinant and native EmTPx proteins. Western blot analysis showed specific detection of recombinant EmTPx and parasite-derived native EmTPx, whereas no specific signal was observed in the negative control (Fig 3D). Immunofluorescence assay and confocal microscopy revealed specific localization of EmTPx in *E. multilocularis* tissues (Fig 3E–3F). In addition, immunohistochemical analysis of paraffin-embedded sections demonstrated strong positive staining in cyst tissues from infected mice, predominantly localized to the cyst wall and partially within cyst fluid, appearing as brown granular deposits (Fig 3G). In contrast, control sections incubated with mouse IgG showed minimal background and no specific staining. These results confirm that the generated EmTPx mAbs specifically recognize native EmTPx protein of *E. multilocularis* without non-specific binding to vector controls.

### Epitope identification

Epitope mapping revealed two distinct linear epitopes recognized by the EmTPx mAbs (Fig 4A; Table 1). As shown in Fig 4B, four mAbs (4E4E3, 1D5D5, 2G2A8, and 3E2D11) reacted with TPx-F3, TPx-F6, TPx-F7, and TPx-F9, defining an epitope within the 174–178 aa region (^174^HPGSK^178^). In contrast, five mAbs (5H8A8, 5F6E6, 5C8B6, 1G2A3, and 5E3A5) consistently recognized TPx-F3, TPx-F6, and TPx-F8, localizing their shared epitope to the 189–193 aa region (^189^SFMSS^193^) (Fig 4C).

**Fig 4:**
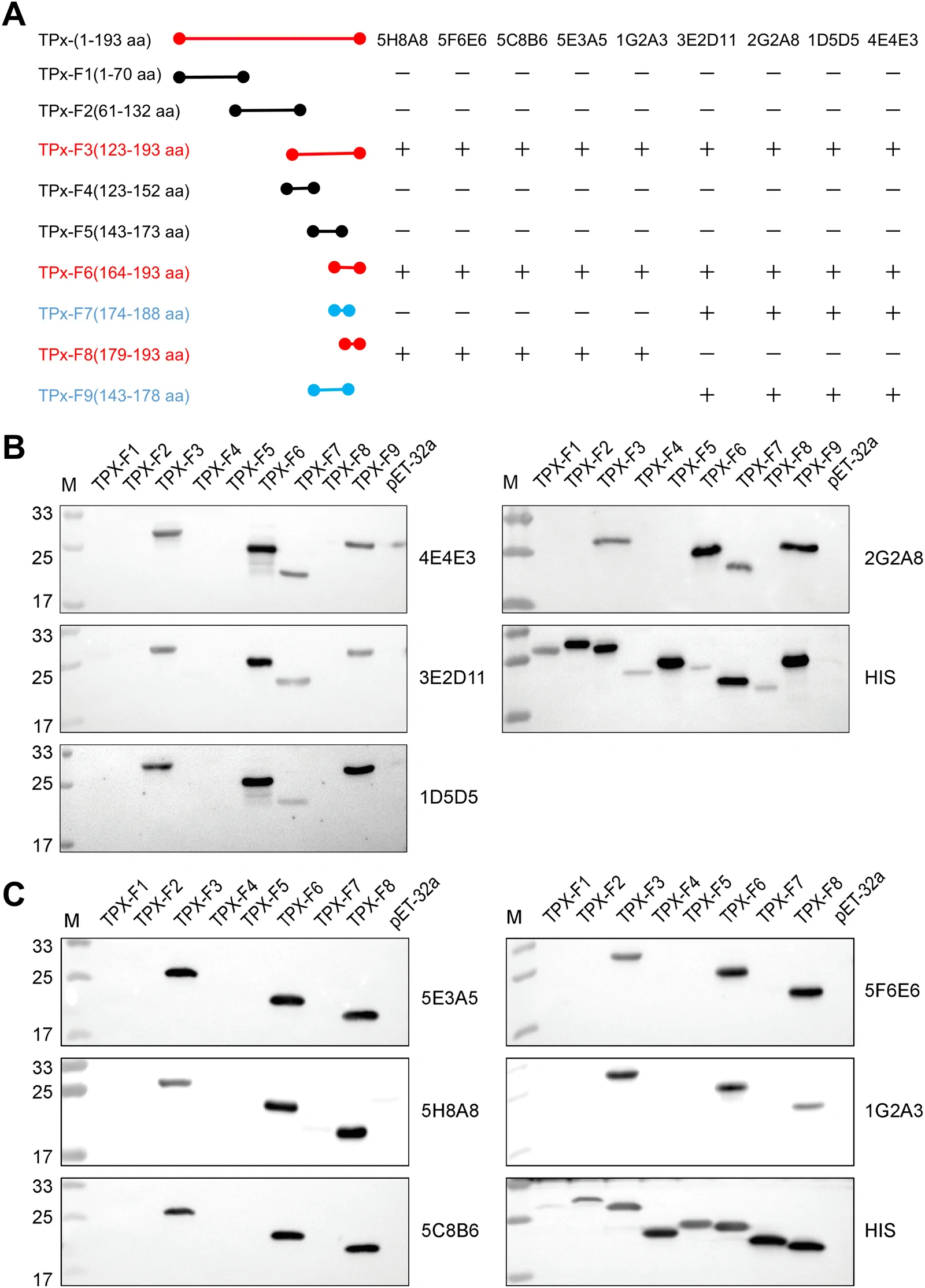
Identification of B‑cell epitopes recognized by EmTPx mAbs. **(A)** Schematic representation of the nine truncated EmTPx fragments. **(B)** Expression of truncated EmTPx fragments (TPx‑F1 to TPx‑F9) in *E. coli* BL21 and Western blot analysis using 1D5D5, 4E4E3, 2G2A8, and 3E2D11 mAbs and anti‑His antibody. Lane M, protein marker; lane 1, 1–70 aa; lane 2, 61–132 aa; lane 3, 123–193 aa; lane 4, 123–152 aa; lane 5, 143–173 aa; lane 6, 164–193 aa; lane 7, 174–188 aa; lane 8, 179–193 aa; lane 9, 143–178 aa; lane 10, pET‑32a empty vector. **(C)** Expression of truncated EmTPx fragments (TPx‑F1 to TPx‑F8) in *E. coli* BL21 and Western blot analysis using 5E3A5, 5F6E6, 5H8A8, 1G2A3, 5C8B6 mAbs and anti‑His antibody. Lane M, protein marker; lane 1, 1–70 aa; lane 2, 61–132 aa; lane 3, 123–193 aa; lane 4, 123–152 aa; lane 5, 143–173 aa; lane 6, 164–193 aa; lane 7, 174–188 aa; lane 8, 179–193 aa; lane 9, pET‑32a empty vector.

### Analysis of B-cell epitopes

Cross-species alignment of EmTPx epitope sequences revealed marked divergence between parasites and hosts in the regions encompassing ^174^HPGSK^178^ and ^189^SFMSS^193^(Fig 5A, 5B). In the ^174^HPGSK^178^ region, *E. multilocularis* and *E. granulosus* display HPGSK and QPGSK, respectively, with complete conservation of core residues, whereas host mammals exhibit KPGSD, with substitutions at both terminal positions. Similarly, in the ^189^SFMSS^193^ region, *E. multilocularis* and *E. granulosus* share an identical sequence (SFMSS), while host species (human, bovine, ovine, canine and fox) consistently present EYFSK. Although homologous TPx proteins from potentially co-infecting parasitic species exhibited overall sequence similarity, the identified EmTPx epitopes showed distinct conservation patterns among different species (S1A- S1C Fig).

To define the structural context of these epitopes, the three-dimensional structure of EmTPx was predicted using AlphaFold3 (pLDDT>90) and visualized in PyMOL. Both epitopes (^174^HPGSK^178^ and ^189^SFMSS^193^) are localized on the protein surface, clearly separated from the hydrophobic core (Fig 5C). Their surface exposure suggests high accessibility, consistent with efficient recognition by antibodies.

**Fig 5:**
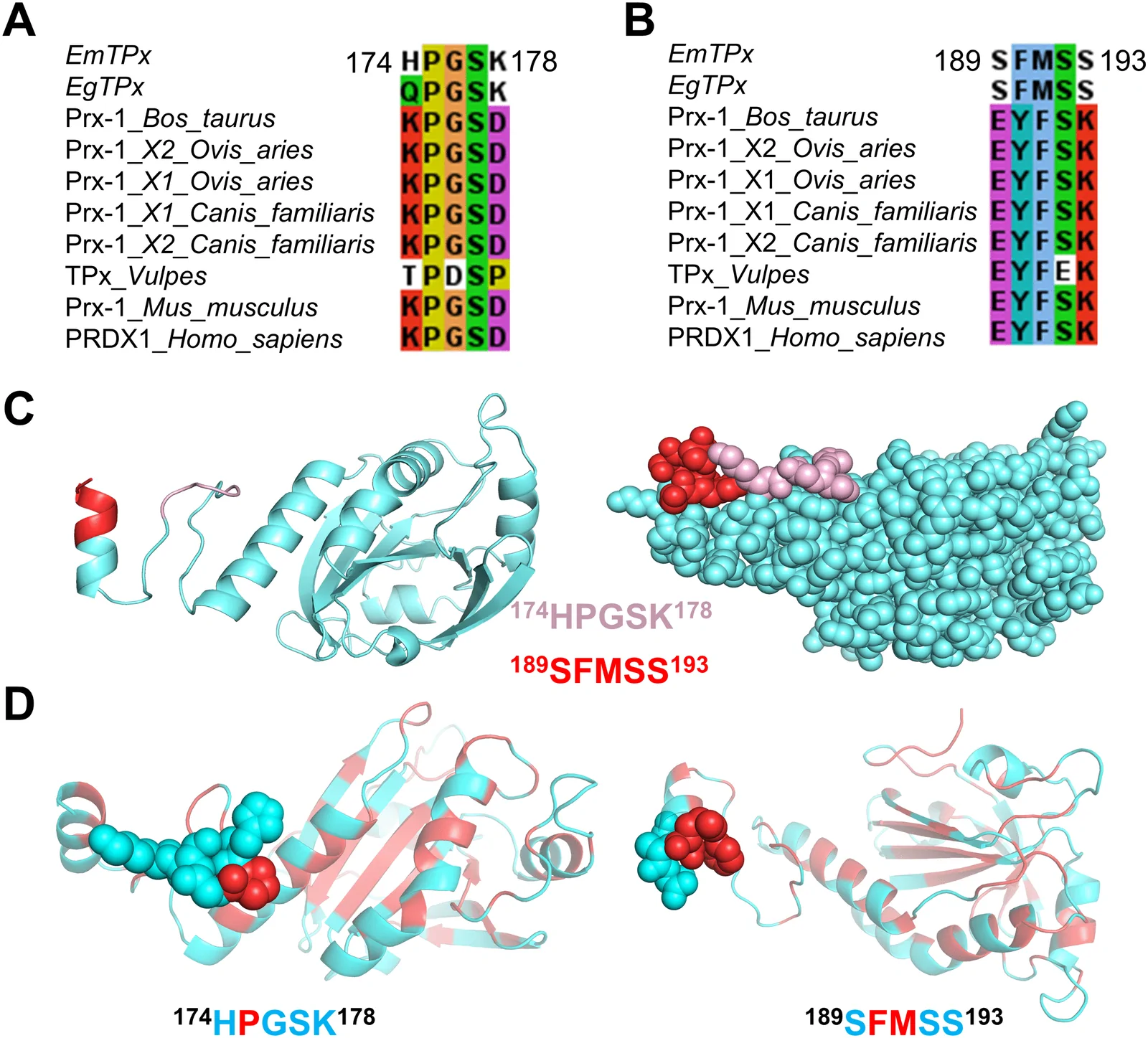
B‑cell epitope identification of EmTPx. **(A)** Sequence alignment of the ^174^HPGSK^178^ epitope across different species. **(B)** Sequence alignment of the ^189^SFMSS^193^ epitope across different species. **(C)** Spatial localization of the two epitopes in the predicted EmTPx structure. The ^174^HPGSK^178^ epitope is shown in pink, and the ^189^SFMSS^193^ epitope is shown in red, displayed in both sphere and cartoon representations. **(D)** Hydrophobicity and hydrophilicity analysis of epitope residues. Hydrophobic residues are shown as red spheres, and hydrophilic residues are shown as cyan spheres.

Structurally, ^174^HPGSK^178^ resides within a flexible loop, whereas ^189^SFMSS^193^ is positioned adjacent to an α-helix (Fig 5C). Loop regions typically exhibit greater conformational flexibility, facilitating antigen-antibody interactions, whereas the helix-proximal epitope remains stably solvent-exposed. Both epitopes display hydrophilic or moderately hydrophilic properties, consistent with their surface localization and characteristic of B-cell epitopes (Fig 5D). Notably, the physicochemical properties of ^174^HPGSK^178^, including its hydrophilicity and positive charge, may favor its exposure on the protein surface and facilitate antibody recognition. Similarly, the amphipathic characteristics of ^189^SFMSS^193^ may influence its interaction with antibodies.

### Establishment and application of EmTPx-based blocking ELISA

Following purification from ascites, SDS‑PAGE analysis showed that each antibody preparation contained only the heavy and light chains, indicating high purity suitable for downstream applications (Fig 6A). Among the nine monoclonal antibodies evaluated using the blocking ELISA (Fig 6B), 1G2A3 exhibited the highest inhibition rate and was therefore selected for the development of the blocking ELISA. Checkerboard titration revealed that an EmTPx coating concentration of 1 μg/mL and a detection antibody dilution of 1:8000 produced the highest inhibition rate, considering the reduced consumption of antigen and antibody, this combination was selected as the optimal working condition for subsequent assays (Fig 6C). As shown in Fig 6D, blocking with 5% skim milk at 37°C for 30 minutes yielded the optimal blocking efficiency. A serum dilution of 1:1 yielded the highest blocking rate (Fig 6E). A sample incubation time of 90 minutes resulted in the highest inhibition rate (Fig 6F), while incubation of the HRP‑conjugated antibody at 37°C for 60 minutes produced the best detection performance (Fig 6G). Finally, a TMB incubation time of 15 minutes generated the highest inhibition rate (Fig 6H), indicating the optimal color development condition.

**Fig 6:**
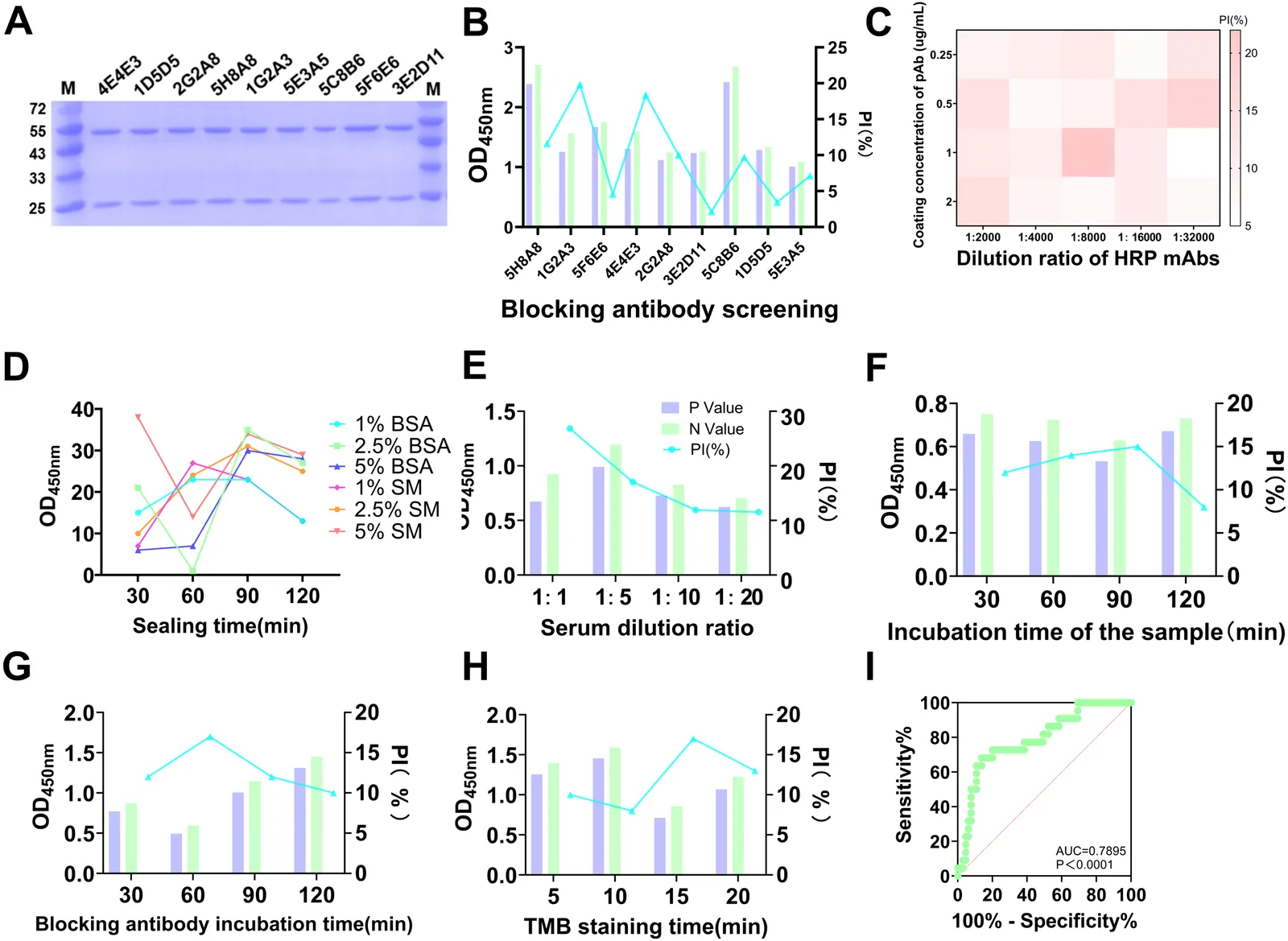
Establishment and optimization of the blocking ELISA. **(A)** SDS‑PAGE analysis of purified mAbs. **(B)** Blocking ELISA using different mAbs as blocking antibodies. **(C)** Optimization of antigen‑coating concentration and HRP‑mAb working dilution. Blocking rates are visualized as a heatmap based on color intensity. **(D)** Optimization of blocking buffer and blocking time**. (E)** Sample dilution ratio. **(F)** Optimization of sample incubation time. **(G)** Optimization of HRP‑mAb incubation time. **(H)** Optimization of TMB substrate color‑development time. Positive values and Negative values are shown as bar graphs, and blocking rates are shown as line plots. **(I)** ROC curve of the blocking ELISA for distinguishing positive and negative sheep serum samples.

Using these optimized parameters, 65 negative sera and 22 positive sera were tested. ROC curve analysis yielded an AUC = 0.7895 (P < 0.0001) (Fig 6I). Based on the maximum Youden index, the optimal cutoff value was determined to be 29.24%, corresponding to a Youden index of 0.5273, with a preliminary diagnostic sensitivity of 72.73% and specificity of 80% (95% CI: 0.6790–0.9000). In the repeated experiments, three positive samples and three negative samples were selected to determine the intra-batch and inter-batch CV, which were all maintained below 10% (Tables 3 and 4).

**Table 3:** Blocking ELISA intra-batch repetitive experiment.

Sample	PI			Mean Value	Standard Deviation	Coefficient of Variation
	1	2	3			
1	31.29%	30.94%	31.11%	0.3111	0.0017	0.56%
2	29.96%	30.36%	31.21%	0.3051	0.0064	2.10%
3	30.25%	29.02%	28.48%	0.2925	0.0091	3.10%
4	20.84%	22.06%	24.77%	0.2256	0.0201	8.91%
5	13.89%	14.12%	15.79%	0.1460	0.0104	7.11%
6	6.03%	6.60%	5.75%	0.0613	0.0043	7.07%

^a^Intra-assay precision was evaluated by analyzing six samples in triplicate within the same plate. The mean, standard deviation, and coefficient of variation were calculated based on the blocking rates, with CV calculated as SD/mean × 100%.

**Table 4:** Blocking ELISA inter-assay repeatability test.

Sample	PI			Mean Value	Standard Deviation	Coefficient of Variation
	1	2	3			
1	30.43%	31.27%	31.06%	0.3092	0.0044	1.41%
2	30.82%	31.01%	29.74%	0.3053	0.0068	2.24%
3	34.15%	34.62%	37.68%	0.3548	0.0192	5.41%
4	5.01%	4.93%	4.68%	0.0487	0.0017	3.50%
5	5.62%	5.94%	5.27%	0.0561	0.0034	5.98%
6	18.91%	18.80%	20.85%	0.1952	0.0115	5.91%

^a^Inter-assay precision was determined by testing the same six samples in triplicate across three independent assays. The mean, standard deviation, and coefficient of variation were calculated based on the blocking rates, with CV calculated as SD/mean × 100%.

### Establishment and application of EmTPx-based sandwich ELISA

Based on differences in antibody isotypes and epitope locations (Table 5 and Fig 5C), a checkerboard titration was performed to identify the optimal antibody pairing for the sandwich ELISA. The combination of 3E2D11 as the capture antibody and 5H8A8 as the detection antibody yielded the highest P/N ratio (Fig 7A) and was therefore selected for subsequent optimization. When the capture antibody concentration was 2 μg/mL and the detection antibody dilution was 1:4000, the P/N ratio reached 31.03 (Fig 7B). Although several combinations showed comparable inhibition rates, 2 μg/mL antigen coating combined with 1:4000 antibody dilution was selected because it provided sufficient signal intensity while reducing antigen and antibody consumption. As shown in Fig 7C, blocking with 5% skim milk at 37°C for 60 minutes produced the highest P/N ratio (38.69), representing the optimal blocking condition. A sample incubation time of 60 minutes resulted in a P/N ratio of 9.85 (Fig 7D), while incubation of the HRP‑conjugated detection antibody at 37°C for 90 minutes yielded a P/N ratio of 13.36 (Fig 7E), indicating the optimal antigen–antibody binding conditions. Finally, a TMB incubation time of 15 minutes produced the highest P/N ratio (Fig 7F), and was therefore selected as the optimal color development duration.

**Table 5:** mAbs subtype epitopes.

mAbs	Heavy Chain	Light Chain	Epitope
5H8A8	IgG1	Kappa	^189^SFMSS^193^
5F6E6	IgG1	Kappa	
1G2A3	IgG2b	Kappa	
5E3A5	IgG1	Kappa	
5C8B6	IgG1	Kappa	
2G2A8	IgG1	Kappa	^174^HPGSK^178^
1D5D5	IgG1	Kappa	
4E4E3	IgG1	Kappa	
3E2D11	IgG2b	Kappa	

mAb, monoclonal antibody; IgG, immunoglobulin G; Kappa, kappa light chain.

^a^The table summarizes the heavy- and light-chain types of the monoclonal antibodies, their corresponding epitopes, and the positions of the identified epitopes within the EmTPx protein structure. Epitope positions are numbered according to the amino acid sequence of EmTPx.

**Fig 7:**
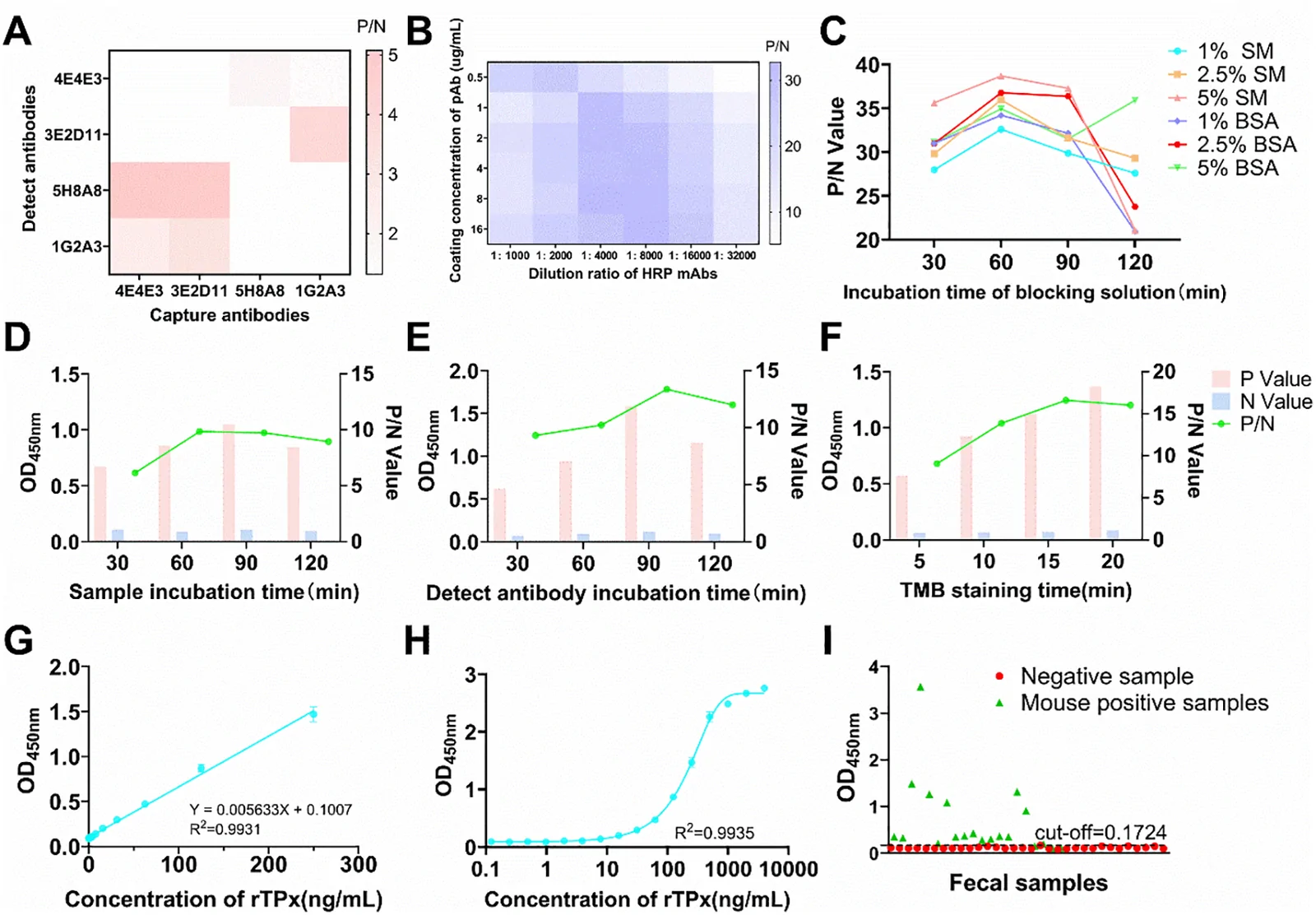
Establishment and optimization of the sandwich ELISA. **(A)** Screening of mAbs with different epitope specificities and isotypes as capture and detection antibody pairs in the sandwich ELISA. **(B)** Optimization of capture‑antibody coating concentration and HRP‑mAb working dilution. P/N values are visualized as a heatmap based on color intensity. **(C)** Optimization of blocking buffer and blocking time. **(D)** Optimization of sample incubation time. **(E)** Optimization of HRP‑mAb incubation time. **(F)** Optimization of the color development time for the TMB substrate. P and N values are displayed as bar charts, and the P/N ratio is presented as a line graph. **(G)** Linear standard curve of the sandwich ELISA for EmTPx detection. **(H)** 4PL standard curve. **(I)** Detection of EmTPx in fecal samples from mice (positive, n = 20; negative, n = 32).

Recombinant EmTPx protein was serially diluted in PBS from 4000 ng/mL to 0.122 ng/mL, and sandwich ELISA was performed under optimized conditions (Fig 7G). OD_450_ readings displayed a strong linear relationship with EmTPx concentration over 0–250 ng/mL (Y = 0.005633X + 0.1007, R^2^ = 0.9931) and were accurately described by a four-parameter logistic (4PL) model (R^2^ = 0.9935, Fig 7H), demonstrating excellent quantitative performance.

Analysis of 32 fecal samples from uninfected mice (Fig 7I) yielded a mean OD_450_ of 0.1164 ± 0.01867, and the cutoff, calculated as the mean plus three times the SD (0.1724), allowed unambiguous classification of samples as positive (OD_450_ > 0.1724) or negative (OD_450_ ≤ 0.1724). These results highlight the assay's sensitivity, reliability, and suitability for accurate quantification of EmTPx in biological samples. Based on the standard curve and the distribution of negative fecal samples, the LOD and LOQ of the assay were estimated to be 12.73 ng/mL and 35.93 ng/mL, respectively.

The optimized dual-epitope sandwich ELISA was subsequently applied to twenty EmTPx‑positive mouse fecal samples, yielding detection rate of 85% (Fig 7I). In the repeated experiments, three positive and three negative samples were selected to determine the intra-batch and inter-batch CV, which were all kept below 10% (Tables 6 and 7). These results indicate that EmTPx-based sandwich ELISA shows promising analytical performance and has potential for non-invasive detection of AE. The successful detection of EmTPx in fecal samples allowed us to delineate a mechanistic model of antigen excretion in intermediate hosts (Fig 8).

**Table 6:** Sandwich ELISA intra-batch repetitive experiment.

Sample	OD450			Mean Value	Standard Deviation	Coefficient of Variation
	1	2	3			
1	0.1098	0.1013	0.1028	0.1046	0.0045	4.34%
2	0.1064	0.1099	0.1036	0.1066	0.0032	2.96%
3	0.1059	0.1058	0.104	0.1052	0.0011	1.02%
4	0.278	0.2974	0.316	0.2971	0.0190	6.39%
5	1.5621	1.4415	1.4028	1.4688	0.0831	5.66%
6	0.9148	0.8582	0.8343	0.8691	0.0413	4.76%

OD450, optical density measured at 450 nm; ^a^Intra-assay precision was assessed by testing six samples in triplicate within the same plate using the sandwich ELISA. The OD450 values from the three replicate measurements were recorded for each sample, and the mean, standard deviation, and coefficient of variation were calculated. The CV was calculated as SD/mean × 100% and was used to evaluate the consistency of the replicate measurements.

**Table 7:** Sandwich ELISA inter-assay repeatability test.

Sample	OD450			Mean Value	Standard Deviation	Coefficient of Variation
	1	2	3			
1	0.1183	0.1106	0.1112	0.1134	0.0043	3.78%
2	0.1097	0.1117	0.1199	0.1138	0.0054	4.75%
3	0.1068	0.1002	0.1004	0.1025	0.0038	3.66%
4	0.4899	0.4848	0.5334	0.5027	0.0267	5.31%
5	0.8581	0.8457	0.8652	0.8563	0.0099	1.15%
6	0.8887	0.8715	0.8699	0.8767	0.0104	1.19%

OD450, optical density measured at 450 nm; ^a^Inter-assay precision was evaluated using three independent experiments performed on different plates. The same six samples were tested in triplicate in each independent experiment, and the corresponding OD450 values were recorded. The mean, standard deviation, and coefficient of variation were calculated from the replicate measurements across the three independent experiments. The CV was calculated as SD/mean × 100% and was used to evaluate the consistency of the assay between independent experiments.

**Fig 8:**
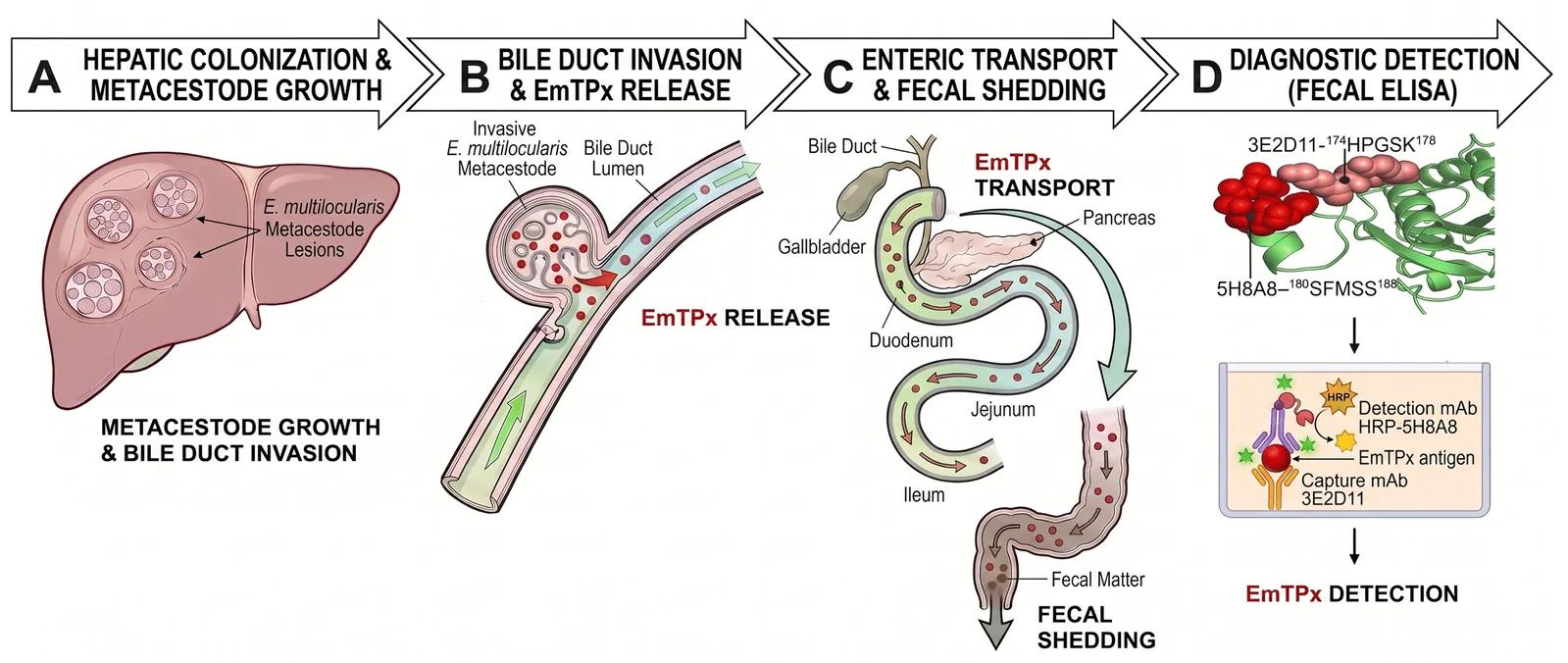
Hepatointestinal trafficking model of EmTPx excretion for coproantigen detection in intermediate hosts. (**A**) *E. multilocularis* metacestodes establish cystic lesions in the liver and release EmTPx through excretory/secretory activities. **(B)** EmTPx enter the biliary tract and reach the intestinal lumen via hepatobiliary excretion. The resulting coproantigen is ultimately shed in feces (**C**) and can be detected non-invasively using the dual-epitope sandwich ELISA developed in this study **(D)**. This Fig was created with sci-draw.com.

## Discussion

Alveolar echinococcosis (AE) remains a serious zoonosis demanding early, accurate diagnosis to guide clinical management and surveillance [24]. Current serology primarily detects host antibodies against antigens such as Em18 and Em2 [7,25], but this strategy is inherently constrained by the host immune status. False negatives frequently occur during early infection or in immunocompromised individuals [26]. Antigen detection, especially through non-invasive sampling, can directly reflect active parasite burden and thus circumvent these limitations. In this study, we conceived a strategy centered on EmTPx, a functionally indispensable, abundantly secreted peroxiredoxin of *E. multilocularis*, and established the first sandwich ELISA for its direct detection in feces. Importantly, the ability to detect EmTPx in fecal samples from experimentally infected mice at an early infection highlights the potential value of this assay for monitoring active infection in intermediate hosts.

The choice of EmTPx as a diagnostic target is supported by several biologically advantageous features. As a core antioxidant enzyme, EmTPx is constitutively expressed across developmental stages to detoxify host-derived reactive oxygen species, while previous proteomic studies have demonstrated its high abundance and strong conservation within the *Echinococcus* genus [27]. Accumulating evidence indicates that TPx proteins in several parasitic helminths are not restricted to intracellular antioxidant functions but can also be actively released into the host environment as E/SPs [28]. Consistent with this, EmTPx exhibited robust immunogenicity and was readily recognized by sera from infected hosts. These characteristics suggest that EmTPx fulfills several key criteria of an ideal diagnostic target, including evolutionary conservation within parasites and sufficient divergence from host homologs.

Crucially, we mapped two linear B‑cell epitopes, namely ^174^HPGSK^178^ and ^189^SFMSS^193^, that are surface-exposed and located within structurally accessible regions. The flexible loop region (^174^HPGSK^178^) may facilitate antibody recognition through its conformational flexibility, while the adjacent helix region (^189^SFMSS^193^) may provide a relatively stable binding interface. These structural features may contribute to efficient antibody–epitope interactions and the high sensitivity of the sandwich ELISA. These epitopes are completely conserved between *E. multilocularis* and *E. granulosus* yet diverge substantially from mammalian TPx homologs, displaying a parasite conserved but host divergent signature. This molecular pattern provides an inherent specificity mechanism that our mAbs cross‑react within the genus while avoiding host TPx interference, minimizing false‑positive background and representing a highly advantageous characteristic for diagnostic target selection [29]. The structural characteristics of these epitopes further provide a plausible explanation for the robust performance of the sandwich ELISA. Their surface accessibility facilitates efficient antibody recognition under native conditions, while their spatial separation permits simultaneous engagement by capture and detection antibodies with minimal steric interference, thereby enhancing antigen capture and signal generation [29,30]. Together, these features provide a structural basis for efficient antigen recognition and assay performance and likely contribute to the sensitivity and reproducibility of the assay.

In this study, EmTPx was successfully detected in fecal samples from experimentally infected rodents, demonstrating that a parasite-derived E/SP from a tissue-dwelling cestode can be identified in feces. This finding is consistent with the possibility that parasite-derived secretory proteins may reach the intestinal lumen through host physiological pathways. Unlike serological assays, which may remain positive long after treatment and therefore poorly reflect ongoing parasite activity [31,32], coproantigen detection has the potential to serve as a more direct indicator of ongoing parasite activity [33], thereby facilitating treatment monitoring, recurrence surveillance, and longitudinal assessment of disease progression. Given that EmTPx is an E/SP during the larval stage of *E. multilocularis*, we propose that EmTPx released by the parasite enters the bile ducts and is subsequently transported to the intestinal lumen of the host via the liver–bile–gut axis, ultimately being excreted in feces. *E. multilocularis* metacestodes reside predominantly within hepatic tissues, and released E/SPs such as EmTPx may access the biliary system through cyst–biliary communications observed in AE lesions. The anatomical plausibility of this hepatointestinal trafficking route is further supported by previous studies describing the close association between hepatic metacestode lesions and the biliary system, including the occurrence of cysto-biliary communications in AE lesions [34]. The detection of parasite antigens in feces has been established using ELISA-based approaches, mainly relying on polyclonal antibodies and fecal samples from definitive hosts. While these assays achieve high specificity (95–98%), undefined epitopes and complex antigen composition can cause batch-dependent variability and reduced sensitivity at low antigen levels [35,36]. Notably, we demonstrated the feasibility of detecting EmTPx antigen in fecal samples from experimentally infected intermediate hosts, supporting coproantigen-based diagnosis in this host group. As a defined antigen, EmTPx enables epitope-specific detection with improved standardization, and its fecal detection in experimentally infected mice provides experimental evidence for hepatointestinal translocation, reinforcing the mechanistic basis for coproantigen detection in AE.

The diagnostic utility of fecal antigen detection has been well established across a broad range of infectious diseases, including *Helicobacter pylori* in bacterial infections, *Giardia/Cryptosporidium* in protozoan infections, and *Fasciola hepatica* in helminthiases [37–39]. By directly detecting pathogen-derived molecules, these assays provide a non-invasive approach for infectious disease diagnosis. For AE, such an approach may be particularly valuable because parasite-derived antigens reflect ongoing biological activity more closely than host antibody responses, potentially enabling earlier recognition of infection. Given the prolonged asymptomatic course of AE and limitations of current serological and imaging-based methods, development of non-invasive markers of active parasite infection remains an important unmet need.

We acknowledge that several limitations should be considered. Cross‑reactivity with other common helminths has not been systematically evaluated, which is essential for field specificity. The temporal kinetics of EmTPx levels during infection, including onset, peak, and clearance after treatment, remain undefined. Our preliminary evaluation employed a limited sample set, and larger, multi‑center cohorts are required to robustly determine sensitivity and specificity [40]. Future studies involving well-characterized clinical samples from different endemic settings and disease stages, particularly samples from hosts infected with other *Echinococcus* species, other cestodes such as *Taenia solium*, and common hepatic parasites such as *Fasciola hepatica* and *Clonorchis sinensis*, will be important to further evaluate the specificity and applicability of EmTPx-based detection in diverse epidemiological contexts. Direct in vivo evidence for the hepatic‑biliary‑intestinal transit of EmTPx, such as tracking studies, remains lacking. Addressing these gaps will be critical for translating this assay into a reliable field‑ready diagnostic.

In summary, by integrating functional target selection, high‑resolution epitope mapping, and dual‑epitope antibody pairing, we have developed a non-invasive fecal antigen detection platform for AE based on EmTPx. The resulting sandwich ELISA provides a promising tool for infection screening and epidemiological surveillance in both intermediate hosts. In particular, for intermediate hosts, direct detection of a constitutively secreted parasite antigen may facilitate earlier recognition of active infection, particularly in settings where access to imaging-based diagnosis is limited. The EmTPx-based coproantigen assay could therefore provide a standardized approach for future evaluation in endemic settings.

## Supporting information

S1 File Supplementary Methods. Antibody Purification from Ascites and Optimization of Blocking and Sandwich ELISAs.
(DOCX)

S1 Fig Sequence comparison of EmTPx with TPx homologs from potential co-infecting parasitic species. **A)** Full-length amino acid sequence alignment of EmTPx and TPx homologs from co-infecting parasitic species. **(B&C)** Comparison of the EmTPx epitope 174–178 **(B)** and 189–193 **(C)** with the corresponding sequences of TPx homologs from co-infecting parasitic species.
(TIF)

S1 Raw Gel Original uncropped Western blot and gel image data corresponding to the experiments presented in this manuscript. S1 Raw Gel for Fig 2. Expression and purification of His‑tagged recombinant EmTPx protein. **(A)** PCR amplification of the EmTPx gene. Lane M, DNA marker; lane 1, PCR product of EmTPx; lane 2, negative control. Lanes marked “X” represented irrelevant experimental results. **(B)** Expression of recombinant EmTPx in *E. coli* under 0.5 mM IPTG induction, analyzed by Western blot using anti‑His monoclonal antibody. Lane M, protein molecular weight marker; lane 1, pET‑28a‑TPx recombinant protein; lane 2, pET‑28a empty vector. **(C)** Protein purification results. Lane M, molecular weight marker; lane 1, flow-through; lanes 2–4, induced whole-cell, supernatant, precipitate; lanes 5–13, 20, 40, 60, 80, 100, 200, 300, 400, 500 mM imidazole elution buffer. S1 Raw Gel for Fig 3. Production and characterization of monoclonal antibodies against EmTPx. **(D)** Western blot analysis of the nine mAbs. Lane M, protein molecular weight marker; lane 1, pET‑28a‑EmTPx recombinant protein; lane 2, pET‑28a empty vector; lane 3, protoscolex lysate. S1 Raw Gel for Fig 4. Identification of B‑cell epitopes recognized by EmTPx mAbs. **(B)** Expression of truncated EmTPx fragments (TPx‑F1 to TPx‑F9) in *E. coli* BL21 and Western blot analysis using 1D5D5, 4E4E3, 2G2A8, and 3E2D11 mAbs and anti‑His antibody. Lane M, protein marker; lane 1, 1–70 aa; lane 2, 61–132 aa; lane 3, 123–193 aa; lane 4, 123–152 aa; lane 5, 143–173 aa; lane 6, 164–193 aa; lane 7, 174–188 aa; lane 8, 179–193 aa; lane 9, 143–178 aa; lane 10, pET‑32a empty vector. Lanes marked “X” represented irrelevant experimental results. **(C)** Expression of truncated EmTPx fragments (TPx‑F1 to TPx‑F8) in *E. coli* BL21 and Western blot analysis using 5E3A5, 5F6E6, 5H8A8, 1G2A3, 5C8B6 mAbs and anti‑His antibody. Lane M, protein marker; lane 1, 1–70 aa; lane 2, 61–132 aa; lane 3, 123–193 aa; lane 4, 123–152 aa; lane 5, 143–173 aa; lane 6, 164–193 aa; lane 7, 174–188 aa; lane 8, 179–193 aa; lane 9, pET‑32a empty vector. Lanes marked “X” represented irrelevant experimental results. S1 Raw Gel for Fig 6. Establishment and optimization of the blocking ELISA. **(A)** SDS‑PAGE analysis of purified mAbs. Lane M, protein marker. Lanes 1–9, 4E4E3, 1D5D5, 2G2A8, 5H8A8, 1G2A3, 5E3A5, 5C8B6, 5F6E6, and 3E2D11, respectively.
(PDF)
