# Supplementary material for: Conserved surface-exposed linear epitopes of Echinococcus multilocularis thioredoxin peroxidase enable non-invasive diagnosis of alveolar echinococcosis in intermediate host
Source: PLoS Negl Trop Dis. 2026 Sep 22;20(9):e0014755. doi: 10.1371/journal.pntd.0014755 (PMC13638047; doi:10.1371/journal.pntd.0014755)
Supplement: Supplementary file 1 [file pntd.0014755.s003.pdf]

Fig 2

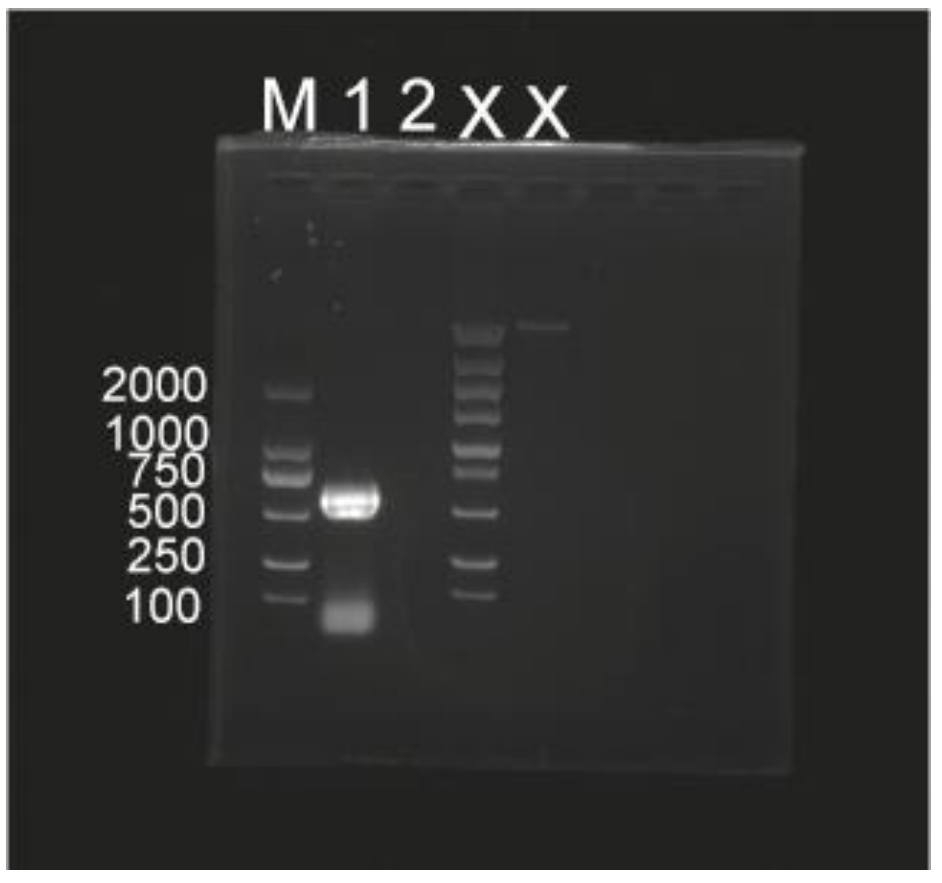

(A) PCR amplification of the EmTPx. Lane M, DNA marker; lane 1, PCR product of EmTPx; lane 2, negative control; Lanes marked "X" represented irrelevant experimental results.

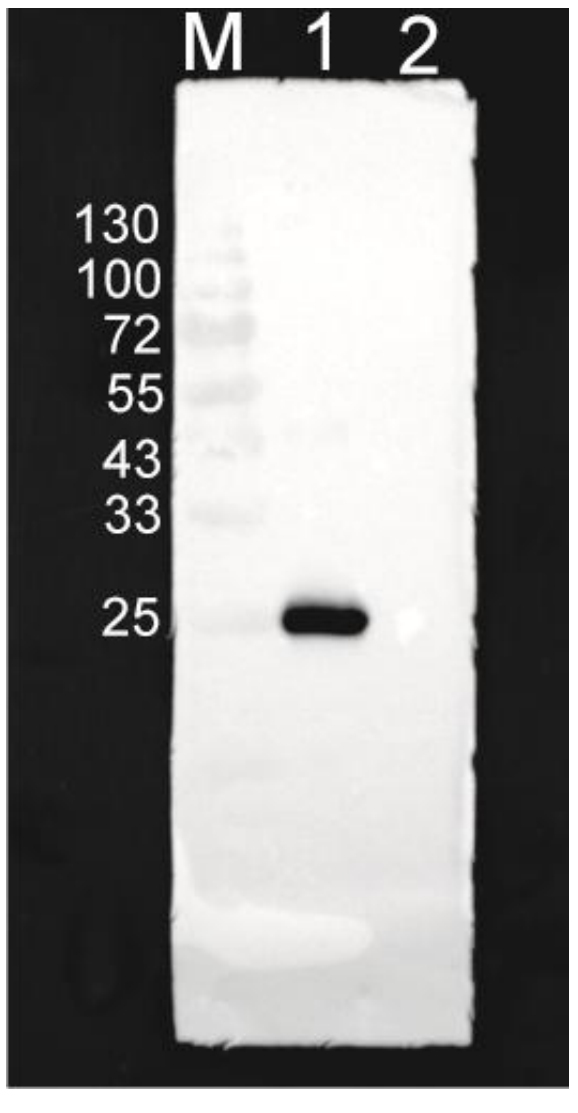

(B) Expression of recombinant EmTPx in *E. coli* under 0.5 mM IPTG induction, analyzed by Western blot using anti His monoclonal antibody. Lane M, protein marker; lane 1, pET-28a TPx recombinant protein; lane 2, pET-28a empty vector.

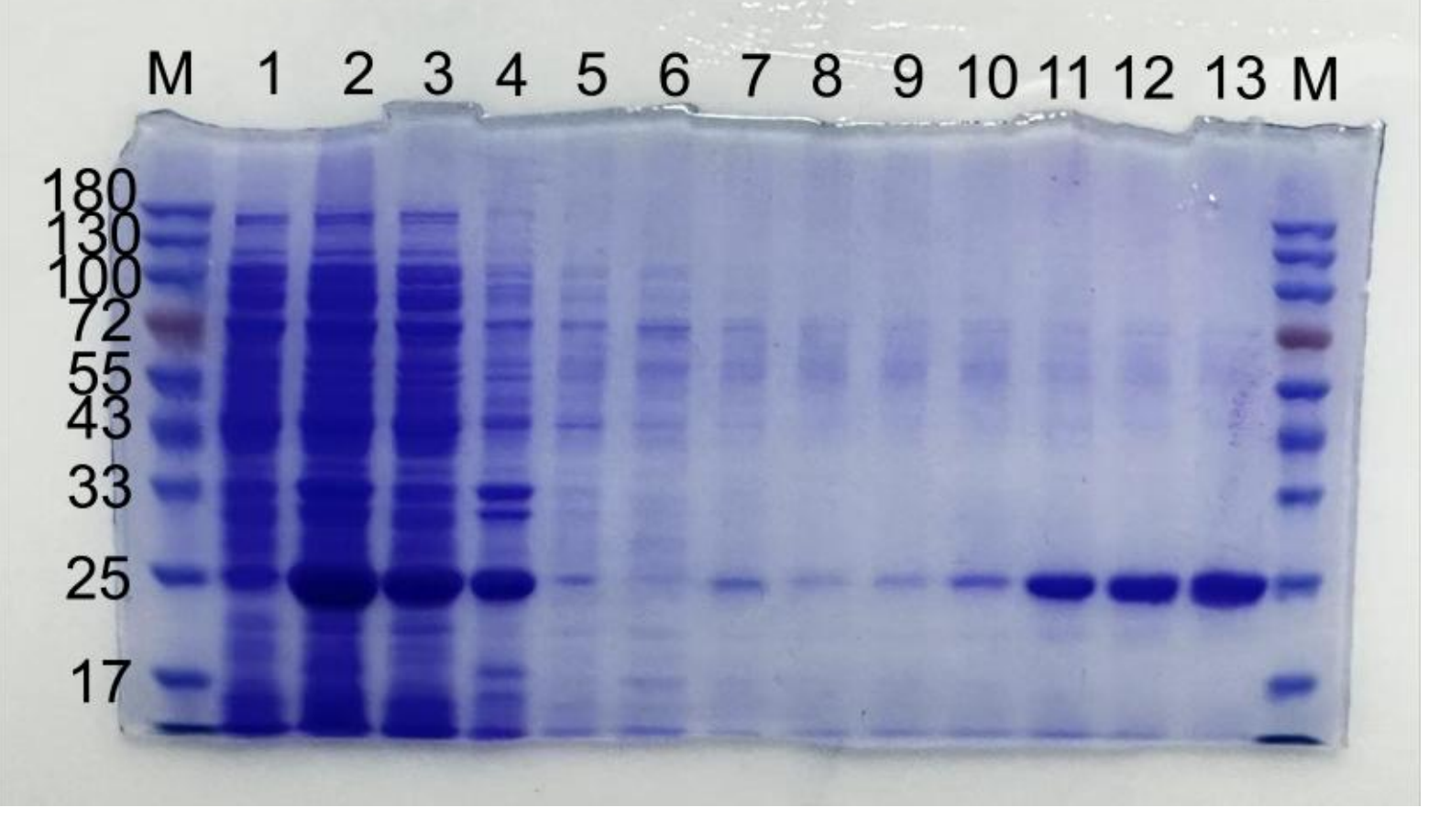

(C) Protein purification results. Lane M, protein marker; 1, Flow-through; 2-4, Induced whole-cell, supernatant, precipitate; 5-13, 20, 40, 60, 80, 100, 200, 300, 400, 500 mM imidazole elution buffer.

Blots were visualized with a TANON 4600 imaging system, while gels were imaged with a TANON 1600B gel imaging system.

Fig 3D

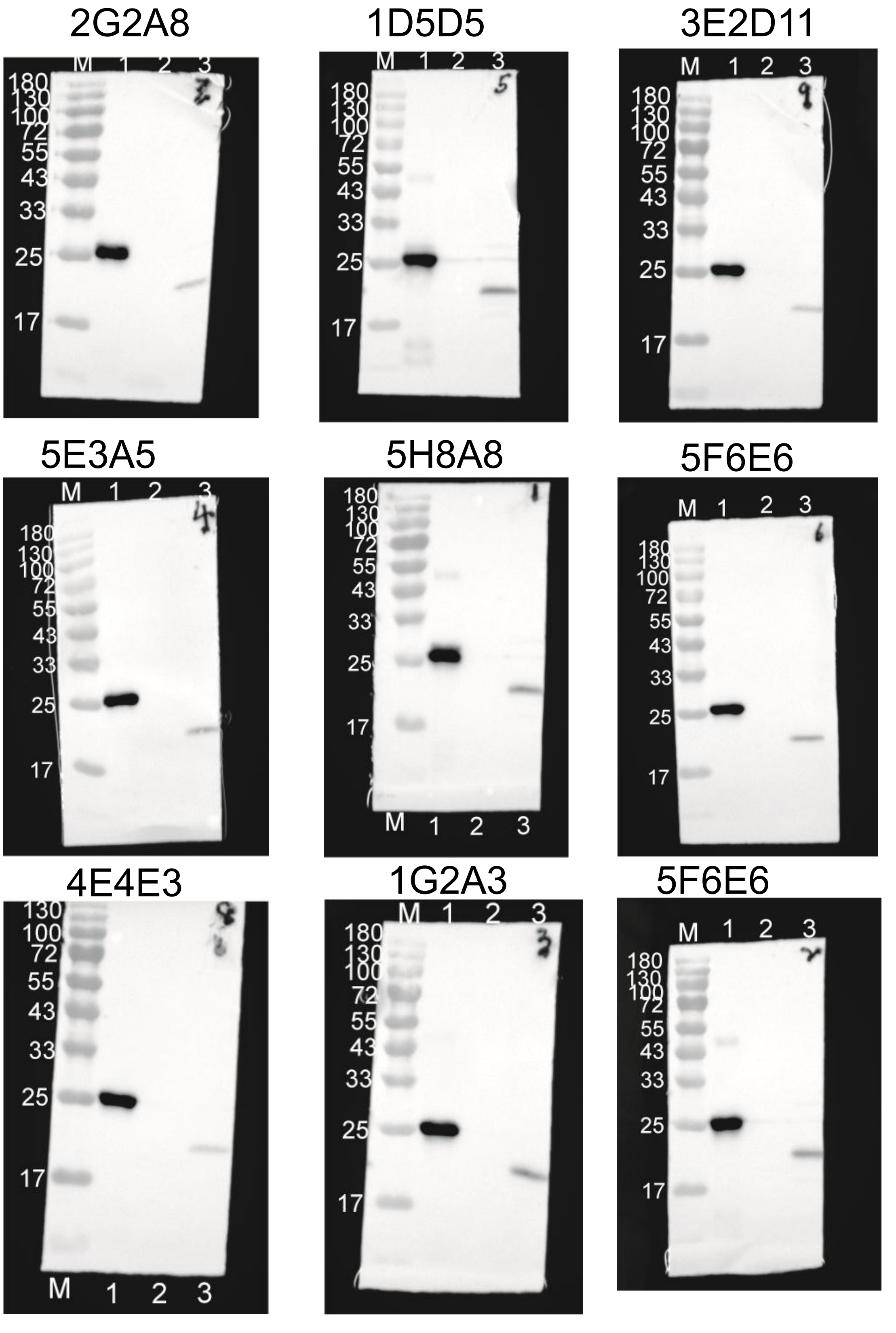

Western blot analysis of the nine mAbs. Each membrane was incubated with the corresponding monoclonal antibody. Lane M, protein marker; lane 1, pET-28a-EmTPx recombinant protein; lane 2, pET-28a empty vector; lane 3, protoscolex lysate.

Fig 4B

4E4E3

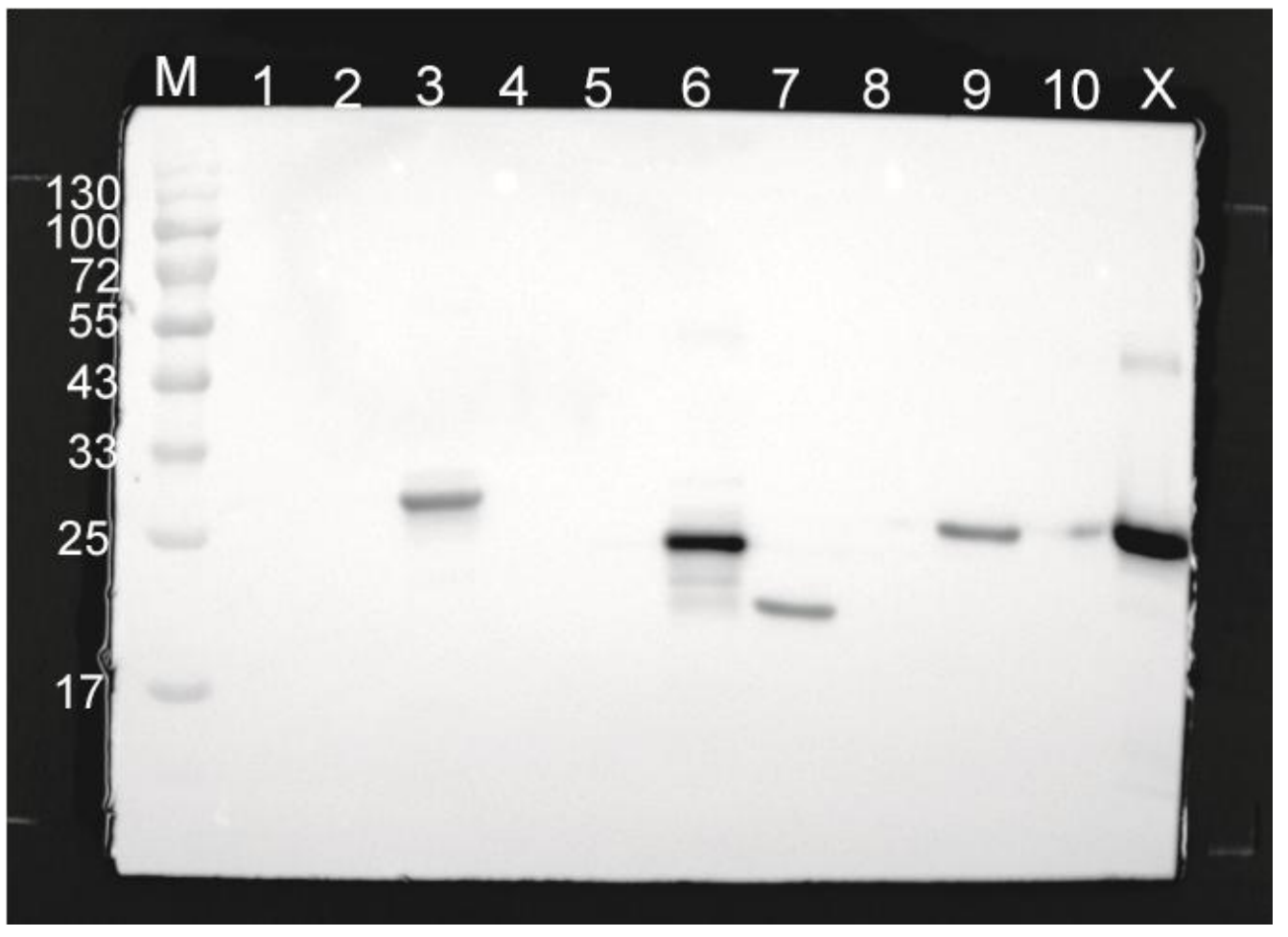

2G2A8

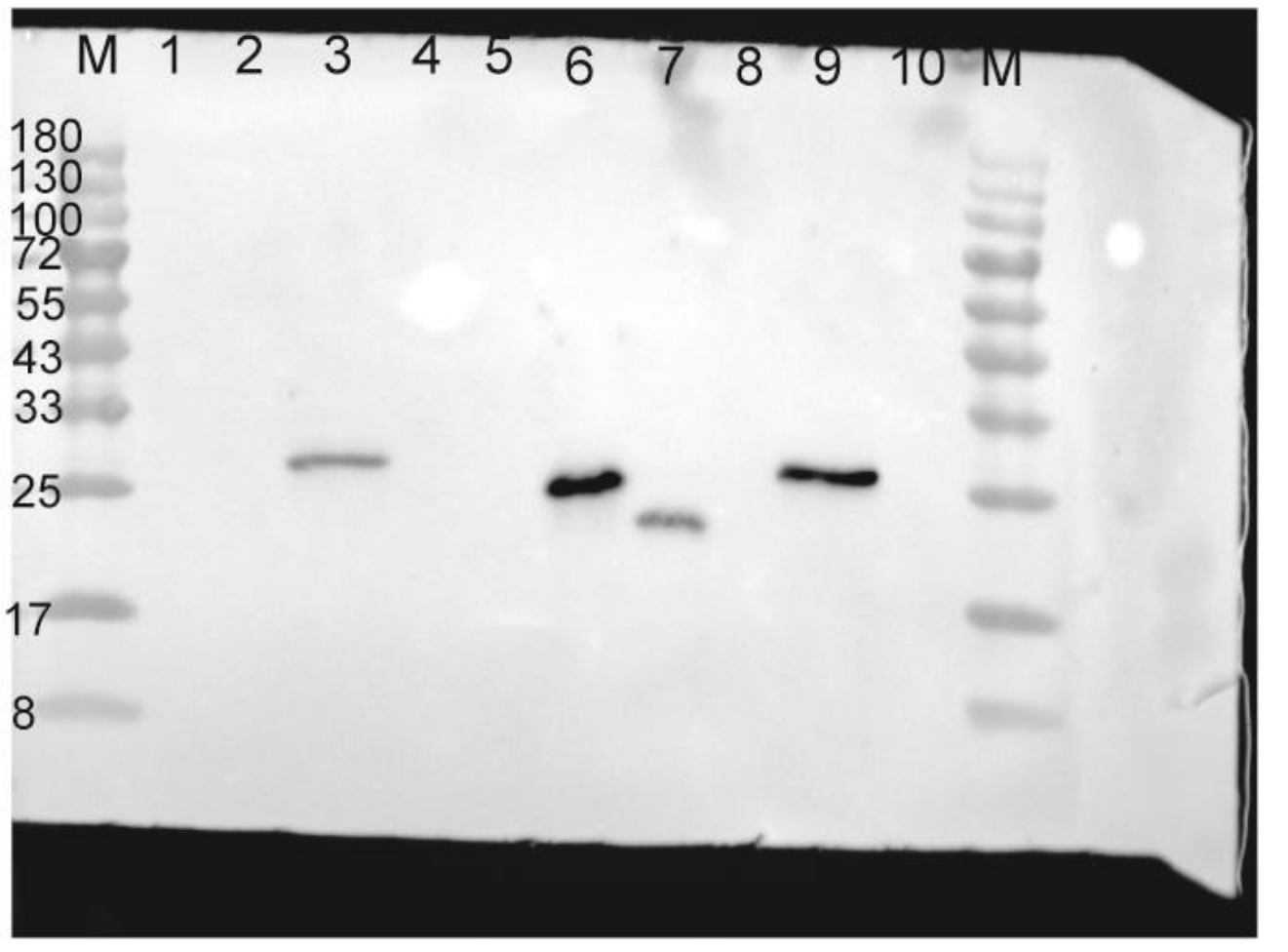

3E2D11

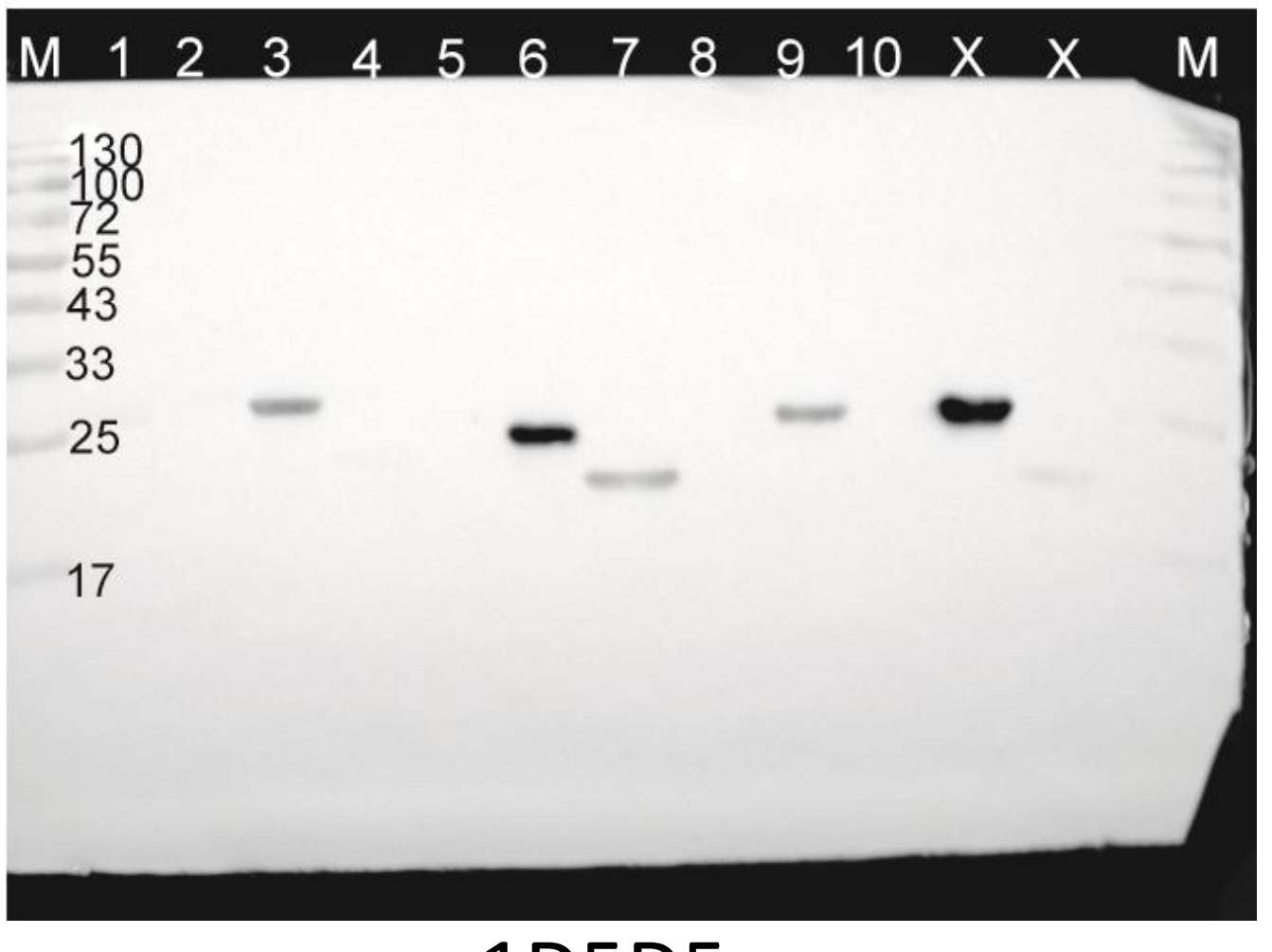

His

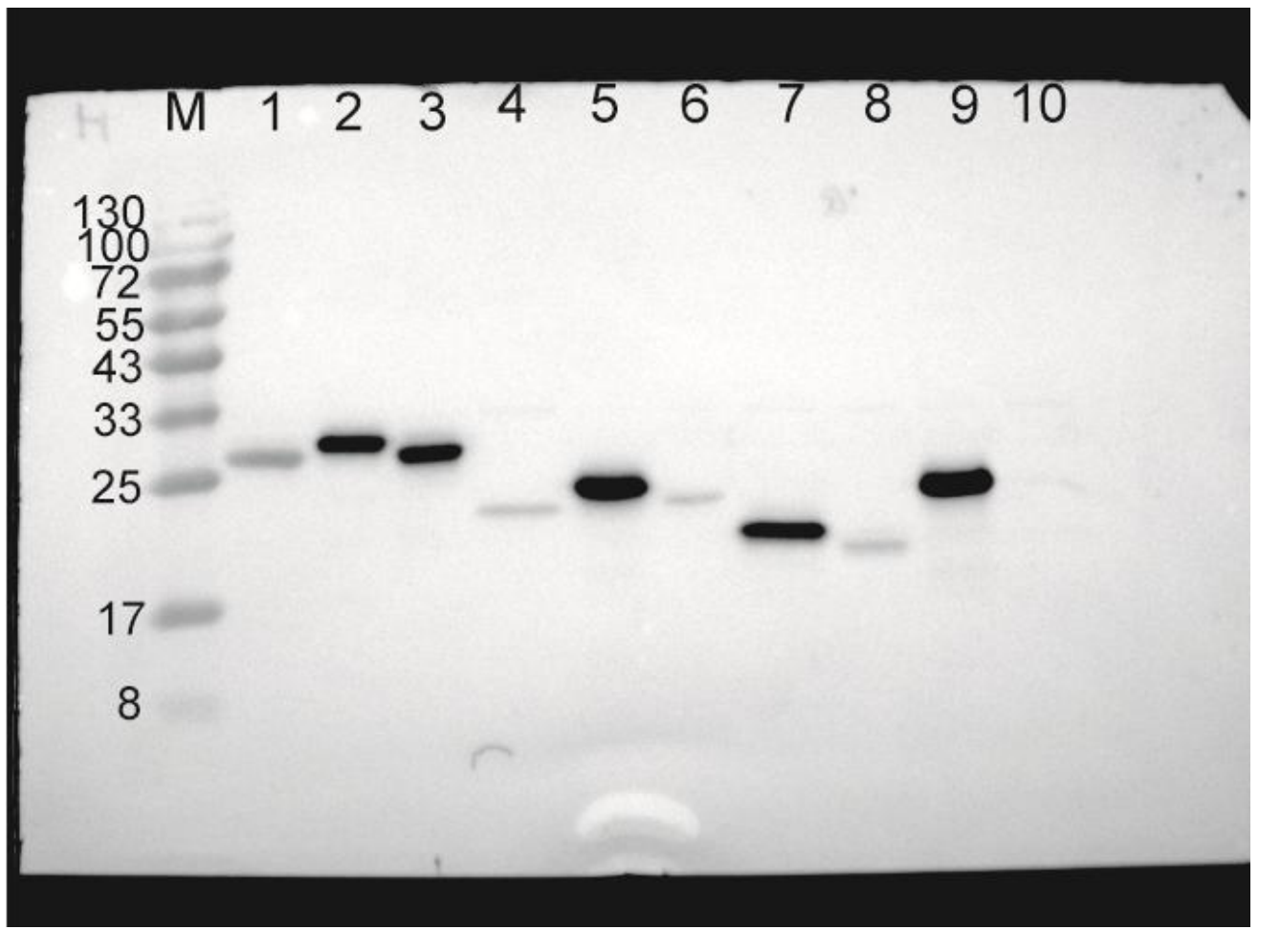

1D5D5

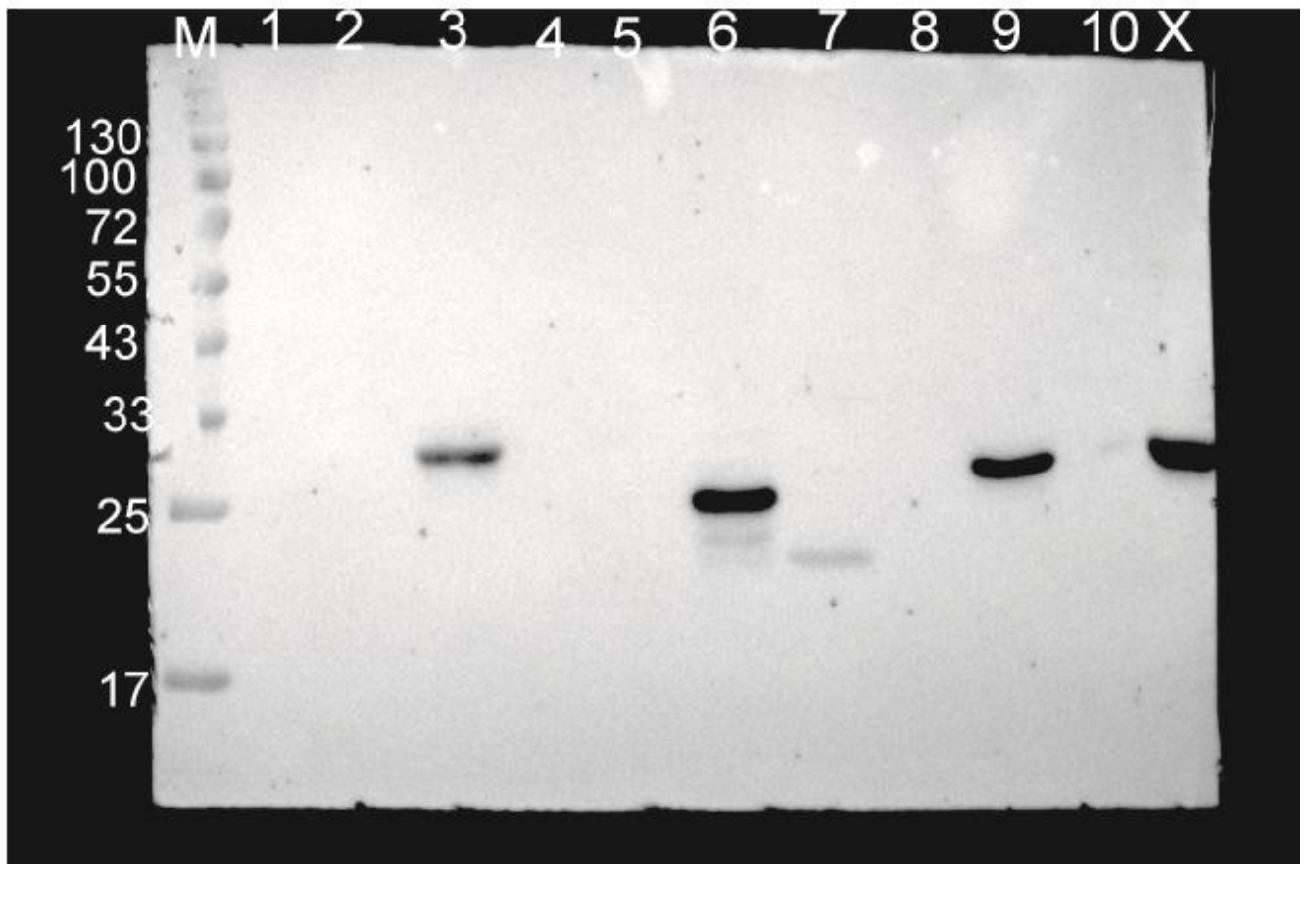

Expression of truncated EmTPx fragments (TPx F1 to TPx F9) in *E. coli* BL21 and Western blot analysis using 1D5D5, 4E4E3, 2G2A8, and 3E2D11 mAbs and anti His antibody. Lane M, protein marker; lane 1, 1–70 aa; lane 2, 61–132 aa; lane 3, 123–193 aa; lane 4, 123–152 aa; lane 5, 143–173 aa; lane 6, 164–193 aa; lane 7, 174–188 aa; lane 8, 179–193 aa; lane 9, 143–178 aa; lane 10, pET-32a empty vector.

Lanes marked “X” represented irrelevant experimental results.

Fig 4C

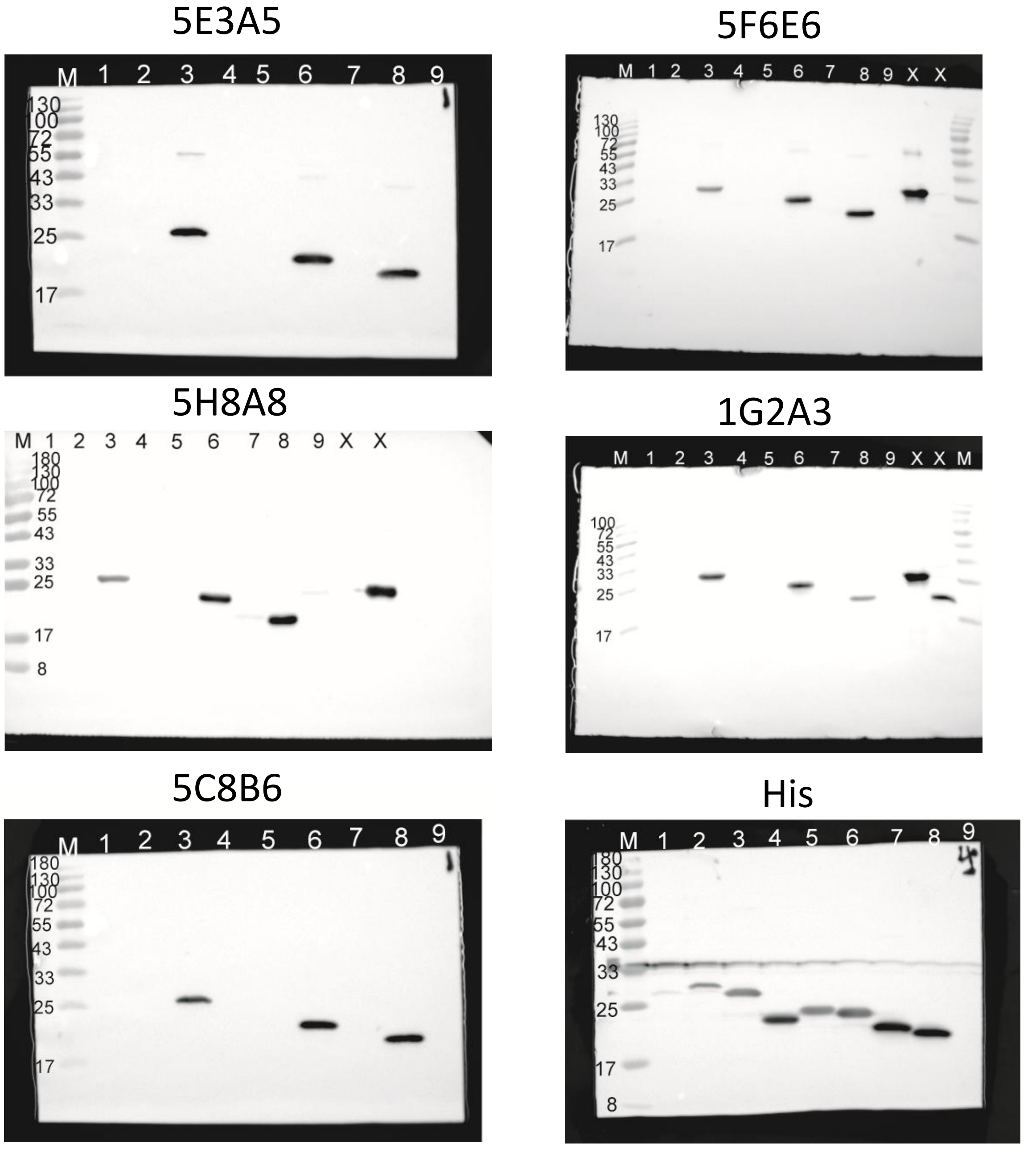

Expression of truncated EmTPx fragments (TPx F1 to TPx F8) in *E. coli* BL21 and Western blot analysis using 5E3A5, 5F6E6, 5H8A8, 1G2A3, 5C8B6 mAbs and anti His antibody. Lane M, protein marker; lane 1, 1–70 aa; lane 2, 61–132 aa; lane 3, 123–193 aa; lane 4, 123–152 aa; lane 5, 143–173 aa; lane 6, 164–193 aa; lane 7, 174–188 aa; lane 8, 179–193 aa; lane 9, pET-32a empty vector.

Lanes marked “X” represented irrelevant experimental results.

Fig 6A

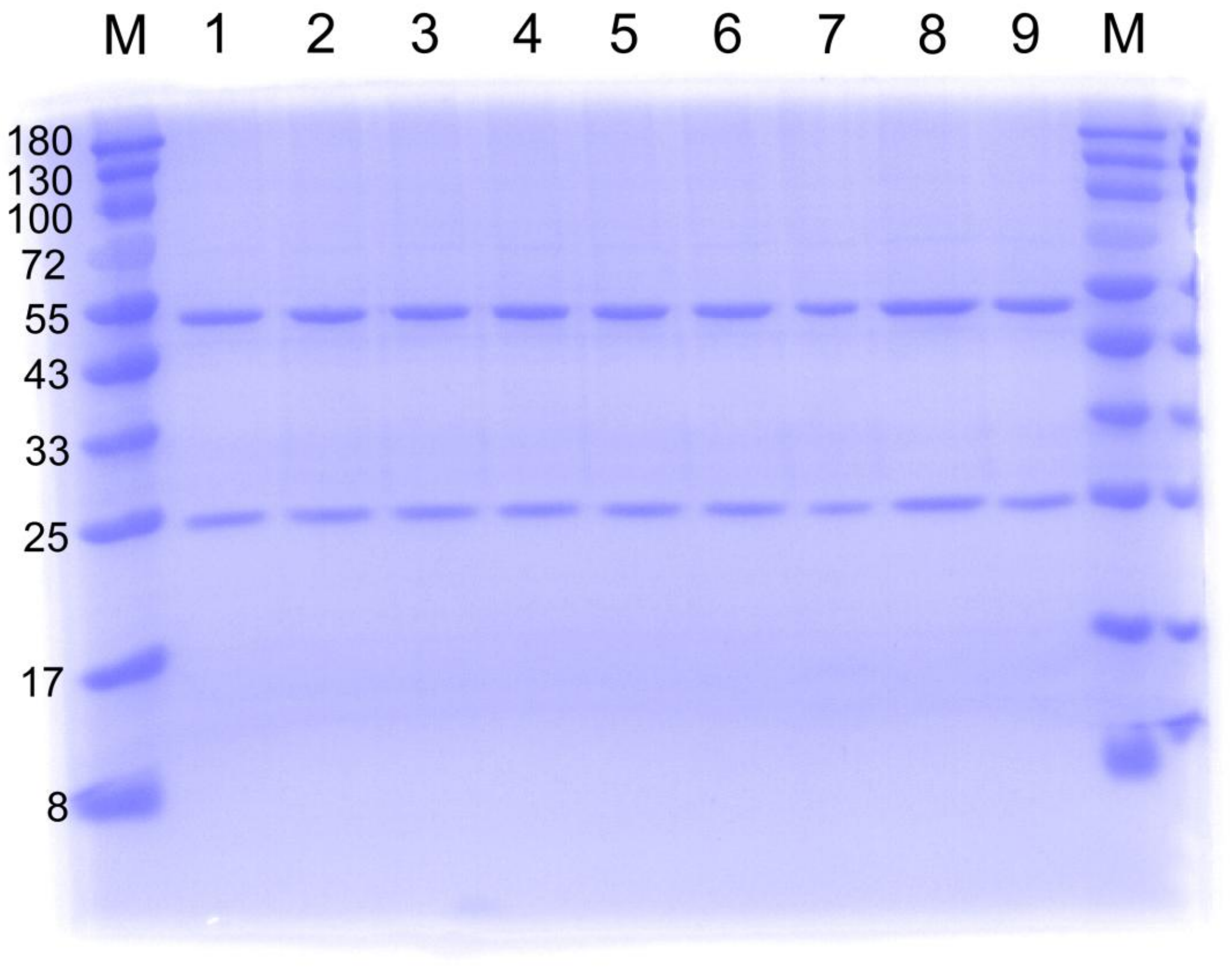

SDS-PAGE analysis of purified mAbs. Lane M, protein marker. Lanes 1–9, 4E4E3, 1D5D5, 2G2A8, 5H8A8, 1G2A3, 5E3A5, 5C8B6, 5F6E6, and 3E2D11, respectively.
