# Supplementary material for: Conserved surface-exposed linear epitopes of Echinococcus multilocularis thioredoxin peroxidase enable non-invasive diagnosis of alveolar echinococcosis in intermediate host
Source: PLoS Negl Trop Dis. 2026 Sep 22;20(9):e0014755. doi: 10.1371/journal.pntd.0014755 (PMC13638047; doi:10.1371/journal.pntd.0014755)
Supplement: Supplementary file 2 [file pntd.0014755.s001.docx]

**Supporting Information**

**S1 File. Supplementary Methods.**

**Antibody Purification from Ascites and Optimization of Blocking and Sandwich ELISAs**

**1. Purification of ascites-derived monoclonal antibodies**

Ascites-derived antibodies were purified using Protein G affinity chromatography. The ascites fluid was diluted 1:5 with equilibration buffer (20 mM Na_2_HPO_4_, 0.15 M NaCl, pH 7.0) and incubated with Protein G resin at 4°C overnight with gentle mixing. The mixture was then loaded onto a gravity-flow column, and the flow through was reapplied to maximize antibody binding. Afterward, the column was washed thoroughly with equilibration buffer, and bound antibodies were eluted using 0.1 M glycine (pH 2.5). The eluate was immediately neutralized by adding 1/10 volume of 1 M Tris HCl (pH 8.5). Purification efficiency was assessed by Coomassie Brilliant Blue staining.

The purified antibodies were dialyzed against 0.01 M carbonate buffer (CB; prepared by dissolving 0.67 g NaHCO_3_ and 0.21 g Na_2_CO_3_ in 1 L of water, pH 9.6) to exchange the buffer system. Finally, the antibodies were concentrated to 2 mg/mL using ultrafiltration tubes.

**2. Screening of antibody pairs for sandwich ELISA**

Two distinct antigenic epitopes of EmTPx were identified, and two monoclonal antibodies were obtained for each epitope based on isotype analysis. To ensure effective antibody pairing for the development of the sandwich ELISA, one representative monoclonal antibody from each isotype group corresponding to the two epitopes was selected, resulting in a total of four monoclonal antibodies (1G2A3, 5H8A8, 3E2D11, and 4E4E3) for subsequent pairing experiments.

To determine the optimal antibody combination for the sandwich ELISA, monoclonal antibodies recognizing different epitopes were evaluated as capture and detection antibodies. ELISA plates were coated with EmTPx‑specific monoclonal antibodies diluted in carbonate coating buffer (pH 9.6) and incubated overnight at 4°C. After three washes with phosphate-buffered saline with Tween-20 (PBST) (PBS containing 0.05% Tween‑20), the plates were blocked with 5% skim milk in PBS at 37°C for 1.5 hours to minimize nonspecific binding.

Following blocking, 100 μL of recombinant EmTPx protein or PBS (negative control) was added to each well and incubated at 37°C for 1.5 hours. After washing with PBST, HRP‑conjugated monoclonal antibodies recognizing different epitopes were added as detection antibodies and incubated at 37°C for 1 hour. The plates were washed again, and 100 μL of TMB substrate solution was added to each well for color development at 37°C for 15 minutes. The reaction was terminated by adding 100 μL of stop solution, and absorbance was measured at 450 nm using a microplate reader.

The ratio of optical density (OD) values between positive wells (EmTPx antigen) and negative wells (PBS) was calculated to identify the optimal capture–detection antibody pair for the sandwich ELISA.

**3. Establishment and optimization of the blocking ELISA method**

After coating ELISA plates with EmTPx protein and incubating overnight at 4°C, the plates were blocked with 5% skim milk at 37°C for 2 hours. Positive and negative sera were diluted 1:1 in PBS, and 100 μL of each diluted serum sample was added to the wells and incubated at 37°C for 2 hours. Following incubation, 100 μL of each of the nine monoclonal antibodies generated in this study (1:2000 dilution) was added to the wells and incubated again at 37°C for 2 hours. After washing, 100 μL of TMB substrate solution was added and allowed to react at 37°C for 15 minutes, followed by the addition of 100 μL stop solution. Absorbance at 450 nm was measured using a microplate reader. The percent inhibition (PI) was calculated as: PI = [(OD450 of negative control - OD450 of test sample) / OD450 of negative control] × 100%. All steps in the ELISA procedure included three washes with PBST (PBS containing 0.05% Tween‑20), each for 5 minutes. The monoclonal antibody 1G2A3, which yielded the highest inhibition rate, was selected for further optimization. Following the same blocking ELISA workflow described above, assay conditions were systematically optimized, including antigen‑coating concentration (0.5, 1, 1.5, 2 μg/mL), detection antibody dilution (1:1000, 1:2000, 1:4000, 1:8000, 1:16000, 1:32000), blocking conditions (1%, 2.5%, 5% BSA or skim milk; incubation time 0.5–2 h at 0.5‑h intervals), serum incubation time (0.5–2 h at 0.5‑h intervals), HRP‑1G2A3 incubation time (0.5–2 h at 0.5‑h intervals), and TMB reaction time (5, 10, 15, 20 min). The optimal condition for each parameter was determined based on the highest inhibition rate.

Using the optimized assay conditions, 22 positive sheep sera and 65 negative sera were tested, and inhibition rates were calculated. The serum samples were collected from farmed sheep by Qinghai University. The infection status of each sample was determined using a commercial *Echinococcus* spp. antibody ELISA detection kit (Zhonglianrui (Beijing) Biotechnology Co., Ltd., Beijing, China; Cat. No. 361425), and samples were classified as positive or negative according to the manufacturer's instructions. The number of serum samples was selected based on the availability of well-characterized samples and was considered sufficient for preliminary evaluation of assay performance by ROC curve analysis. Receiver operating characteristic (ROC) curves were generated using GraphPad Prism 10.4.0. The optimal cutoff value, sensitivity, and specificity were determined using the maximum Youden index, calculated as sensitivity + specificity − 1. The inhibition rate corresponding to the maximum Youden index was defined as the optimal cutoff.

**Supplementary Figure Captions**

**S1 Fig. Sequence comparison of EmTPx with TPx homologs from potential co-infecting parasitic species.**

**(A)** Full-length amino acid sequence alignment of EmTPx and TPx homologs from co-infecting parasitic species. **(B&C)** Comparison of the EmTPx epitope 174–178 **(B)** and 189–193 **(C)** with the corresponding sequences of TPx homologs from co-infecting parasitic species.
